# Machine learning model for cardiovascular disease prediction in patients with chronic kidney disease

**DOI:** 10.3389/fendo.2024.1390729

**Published:** 2024-05-28

**Authors:** He Zhu, Shen Qiao, Delong Zhao, Keyun Wang, Bin Wang, Yue Niu, Shunlai Shang, Zheyi Dong, Weiguang Zhang, Ying Zheng, Xiangmei Chen

**Affiliations:** ^1^ Department of Nephrology, First Medical Center of Chinese PLA General Hospital, National Key Laboratory of Kidney Diseases, National Clinical Research Center for Kidney Diseases, Beijing Key Laboratory of Kidney Diseases Research, Beijing, China; ^2^ School of Clinical Medicine, Guangdong Pharmaceutical University, Guangzhou, China; ^3^ Medical Innovation Research Division of Chinese PLA General Hospital, Beijing, China; ^4^ National Engineering Research Center of Medical Big Data, PLA General Hospital, Beijing, China

**Keywords:** chronic kidney disease, cardiovascular disease, electronic medical records, prediction model, machine learning

## Abstract

**Introduction:**

Cardiovascular disease (CVD) is the leading cause of death in patients with chronic kidney disease (CKD). This study aimed to develop CVD risk prediction models using machine learning to support clinical decision making and improve patient prognosis.

**Methods:**

Electronic medical records from patients with CKD at a single center from 2015 to 2020 were used to develop machine learning models for the prediction of CVD. Least absolute shrinkage and selection operator (LASSO) regression was used to select important features predicting the risk of developing CVD. Seven machine learning classification algorithms were used to build models, which were evaluated by receiver operating characteristic curves, accuracy, sensitivity, specificity, and F1-score, and Shapley Additive explanations was used to interpret the model results. CVD was defined as composite cardiovascular events including coronary heart disease (coronary artery disease, myocardial infarction, angina pectoris, and coronary artery revascularization), cerebrovascular disease (hemorrhagic stroke and ischemic stroke), deaths from all causes (cardiovascular deaths, non-cardiovascular deaths, unknown cause of death), congestive heart failure, and peripheral artery disease (aortic aneurysm, aortic or other peripheral arterial revascularization). A cardiovascular event was a composite outcome of multiple cardiovascular events, as determined by reviewing medical records.

**Results:**

This study included 8,894 patients with CKD, with a composite CVD event incidence of 25.9%; a total of 2,304 patients reached this outcome. LASSO regression identified eight important features for predicting the risk of CKD developing into CVD: age, history of hypertension, sex, antiplatelet drugs, high-density lipoprotein, sodium ions, 24-h urinary protein, and estimated glomerular filtration rate. The model developed using Extreme Gradient Boosting in the test set had an area under the curve of 0.89, outperforming the other models, indicating that it had the best CVD predictive performance.

**Conclusion:**

This study established a CVD risk prediction model for patients with CKD, based on routine clinical diagnostic and treatment data, with good predictive accuracy. This model is expected to provide a scientific basis for the management and treatment of patients with CKD.

## 1 Introduction

Chronic kidney disease (CKD) affects more than 10% of the global population and is closely associated with increases in the incidence and mortality rates of cardiovascular disease (CVD) (1, 2). Therefore, effective prediction and management of the cardiovascular risk in this group are of paramount importance (3).

However, there are few established prediction tools developed specifically for this population, and classical models, such as the Framingham prediction model and the Systematic Coronary Risk Evaluation (SCORE) tool, perform poorly in patients with CKD (4–6). Hence, predicting the risk of cardiovascular events in patients with CKD is an important research area that still requires refinement and the development of more accurate and reliable prediction tools tailored for patients with CKD.

After conducting a literature review, we identified several modeling techniques commonly used in predictive modeling tasks. These include Logistic Regression (LR), Cox Model, Support Vector Machine (SVM), Random Forest (RF), K-Nearest Neighbor (KNN), Extreme Gradient Boosting (XGBoost), and Back propagation Neural Network (BPNN). Of note, XGBoost has emerged as a prevalent machine learning method that has demonstrated effective outcomes in various risk prediction models.Zelnick et al. (7) used a predictive model developed using gradient boosting machines, which demonstrated superior performance in forecasting atrial fibrillation events among patients with CKD compared to previously published predictive models.

This study utilized data from patients’ medical records to identify risk factors associated with cardiovascular events in patients with CKD. Subsequently, machine learning was employed to construct artificial intelligence models to predict disease occurrence and assist clinicians in the timely detection of cardiovascular events.

## 2 Materials and methods

### 2.1 Study population

This was a single-center retrospective study of data sourced from the electronic medical record system of a large tertiary hospital for inpatients with CKD at the Chinese People’s Liberation Army general Hospital(PLAGH). Data were collected from patients who received treatment at the Nephrology Department of the PLAGH between January 1, 2015, and December 31, 2020, totaling 8,894 cases. The inclusion criteria were as follows (1): diagnosis of CKD according to the 2012 Kidney Disease: Improving Global Outcomes guidelines or a clinical diagnosis of CKD; (2) age ≥18 years; and (3) complete data on key clinical indicators including creatinine and routine urine indicators. Patients with acute kidney failure were excluded.

This study was approved by the Ethics Committee of the General Hospital of the Chinese People’s Liberation Army (S2021–696-01). The study was conducted in accordance with the Declaration of Helsinki and was approved by the ethics committee, the informed consent may be exempted from signing.

### 2.2 Definitions

#### 2.2.1 Study outcome

CVD was defined as a composite of cardiovascular events, including coronary heart disease (coronary artery disease, myocardial infarction, angina pectoris, and coronary artery revascularization), cerebrovascular disease (hemorrhagic stroke and ischemic stroke), death from all causes (cardiovascular death, non-cardiovascular death, and unknown cause of death), congestive heart failure, and peripheral artery disease (aortic aneurysm, aortic, or other peripheral arterial revascularization) (8–10). A cardiovascular event was defined as a composite outcome of multiple cardiovascular events, as determined by reviewing medical records.

#### 2.2.2 Other definitions

The specific definition of CKD depended primarily on the pathological diagnosis of biopsy findings in the medical records, on a clinical diagnosis by a nephrologist, or according to the Kidney Disease Outcomes Quality Initiative (KDOQI) guidelines, which define renal injury as an estimated glomerular filtration rate (eGFR)<60 mL/min/1.73 m² for 3 months or more. Renal injury was defined as the presence of pathological abnormalities or injury markers, including abnormal blood or urine test results, or imaging findings. The eGFR was calculated using the Chronic Kidney Disease Epidemiology Collaboration equation (11).

### 2.3 Clinical data extraction

We collected patients’ demographic information, vital signs, and clinical data, including the following: age, sex, history of hypertension, C-reactive protein, serum creatinine (SCr), eGFR, blood urea nitrogen (BUN), total cholesterol, triglycerides, high-density lipoprotein (HDL), low-density lipoprotein, serum cystatin C, serum albumin, hemoglobin, blood potassium, blood sodium, blood calcium, blood chlorine, blood phosphorus, blood magnesium, plasma D-dimer, interleukin 6 activated partial thromboplastin time, prothrombin time, and medication treatment measures, including whether antiplatelet drugs were taken. The 24-h urine protein, body mass index (BMI), neutrophil and lymphocyte ratio, monocyte count/lymphocyte ratio, platelet count/lymphocyte ratio, and platelet count × (neutrophil/lymphocyte count) were also calculated using information from the medical records.

### 2.4 Statistical analysis

Data were computed and statistically analyzed using software SPSS version 26, R software version 4.3.2, and Python version 3.4. Variables with >25% missing values were excluded, and missing data were imputed using the KNN. For continuous variables, comparisons were made using Student’s t-test (for normally distributed variables) or the Wilcoxon rank-sum test (for non-normally distributed variables). Continuous variables conforming to normal or approximately normal distributions were expressed as mean ± standard deviation and were compared using the t-test. Continuous variables not conforming to normal distributions were expressed as median (M) (quartile 1 [Q1], quartile [Q3]) and were compared using the Mann–Whitney U test. Categorical variables were described using counts (%), and comparisons were made using the chi-square test. A P-value<0.05 was considered significant.

### 2.5 Data augmentation

Data augmentation was used to balance the number of patients with and without CVD, with 6,640 cases each. To address data imbalance, we employed the synthetic minority over-sampling technique (12) for data augmentation.

### 2.6 Model construction

The dataset was divided into training and testing sets in an 8:2 ratio. Least absolute shrinkage and selection operator (LASSO) regression analysis was used to select variables that could predict CVD risk. After a thorough literature search, we carefully selected the currently used modeling methods. Seven models were built using machine learning including LR, Naive Bayes (NB), KNN, XGBoost, RF, and Back propagation neural network (BPNN) to evaluate the model performance.

### 2.7 Evaluation metrics for machine learning

Receiver operating characteristic (ROC) curves were drawn to assess the model performance using the accuracy, sensitivity, specificity, F1-score (13) and area under the curve (AUC) as indicators to evaluate the model’s ability to predict cardiovascular events. The formulas for the model evaluation index are as follows: where TP represents the number of true positives; FP, number of false positives; FN, number of false negatives; and TN, number of true negatives.


$$Accuracy\;=\frac{TP\;+\;TN}{TP\;+\;TN\;+\;FP\;+\;FN}$$

$$Sensitivity/recall\;=\;\frac{TP}{TP\;+\;FN}$$

$$Specificity\;=\frac{TN}{TN+FP}$$

$$Precision=\frac{TP}{TP+FP}$$

$$F1-score\;=\frac{2\;\times \;\left\{(Precision*Recall\right)}{\left(Precision+Recall)\right\}}$$

These indicators were used to validate the results, evaluate their ability to predict cardiovascular events, and select the best model.

### 2.8 Model interpretation

Shapley Additive explanations (SHAP) was used to interpret the model results. SHAP (14) values are used to explain the output of any machine learning model by quantifying the impact of each feature on the prediction.

## 3 Results

### 3.1 Study population

**Figure 1:**
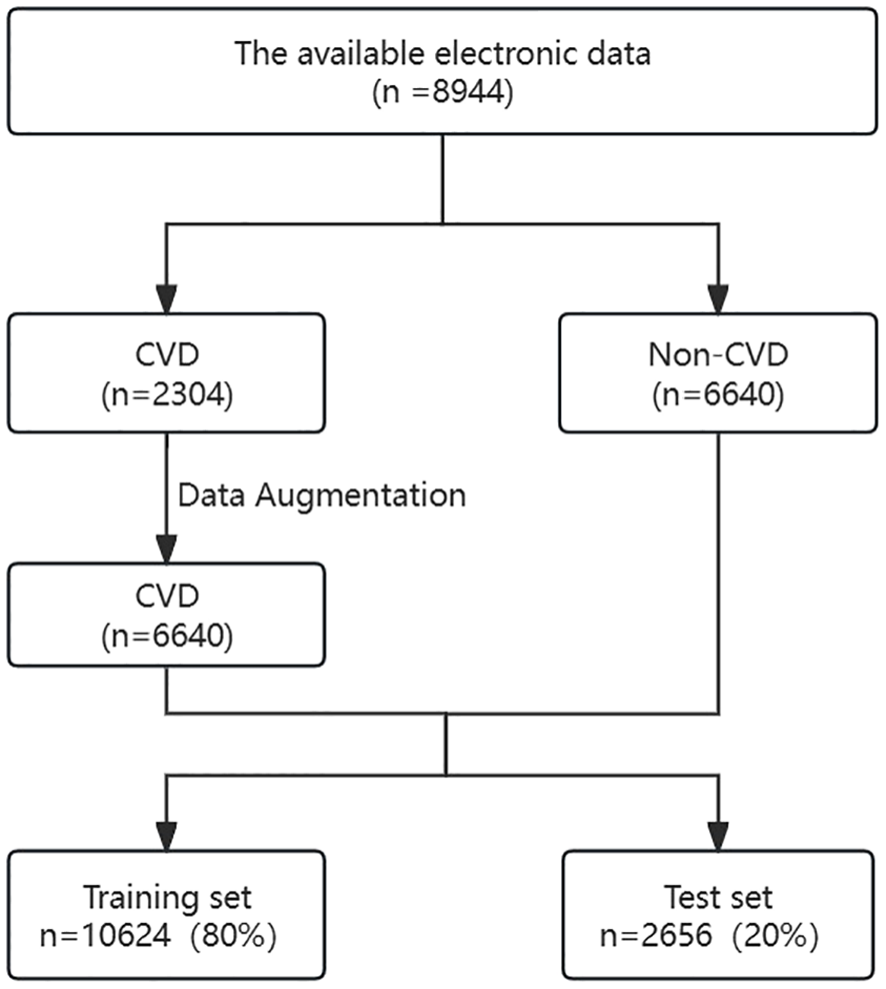
Flowchart of participant screening. CKD, chronic kidney diseases; CVD, cardiovascular disease.

Data from 8,894 patients with CKD were collected; patients were divided based on the presence (n=2,304) or absence (n=6,640) of CVD. As there was an imbalance in the data, data augmentation was used to balance the number of patients with or without CVD to 6,640 cases each. The dataset was divided into a training set (n=10,624) and a testing set (n=2,656) at an 8:2 ratio. Data collected from the training dataset were used to evaluate important variables related to CVD and to establish prediction models. The data from the test set was utilized to assess the performance of the prediction models trained on the training set. The data collection process is illustrated in **Figure 1**.

### 3.2 Clinical characteristics of the included patients

As shown in **Table 1**, a total of 13,280 patients were enrolled in the study following data augmentation. The median age of the participants was 52 years. Among them, 69.1% were male, and 30.9% were female. The incidence of composite CVD events was 50% (6640/13280), with 6,640 patients reaching the outcome. The average age of patients with CVD was significantly higher than that of patients without cardiovascular disease. BMI and prevalence of hypertension were higher in the CVD group than in the non-CVD group. The proportion of males was significantly higher in the CVD group. Nearly all laboratory indicators including hemoglobin, SCr, eGFR, 24-h urinary protein, HDL, BUN, and inflammatory markers such as the neutrophil-to-lymphocyte ratio were significantly different between the CVD and non-CVD groups (P<0.05).

**Table 1:** Clinical characteristics of patients following data augmentation.

Number	ALL(n=13280)	Non-CVD(n=6640)	CVD(n=6640)	P-Value
AGE(years)	52.0 (40.0,62.0)	44.0 (32.8,54.0)	57.9 (50.0,65.3)	<0.001
Gender, n (%)				
Female	4107 (30.9)	2651 (39.9)	1456 (21.9)	<0.001
Male	9173 (69.1)	3989 (60.1)	5184 (78.1)	
BMI(kg/m2)	25.2 (22.8,27.7)	24.8 (22.2,27.5)	25.6 (23.3,27.9)	<0.001
Hypertension, n(%)	9871 (74.3)	3971 (59.8)	5900 (88.9)	<0.001
Hb (g/L)	114.3 (92.8,134.0)	120.0 (97.0,139.0)	108.1 (89.8,129.0)	<0.001
Scr (umol/L)	150.5 (85.6,403.4)	115.9 (77.4,286.1)	206.5 (101.1,463.2)	<0.001
eGFR, mL/min/1.73 m^2^	44.6 (13.0,87.8)	62.5 (20.4,99.8)	30.8 (11.0,70.1)	<0.001
Urine protein (g/day)	2.5 (1.2,4.4)	2.1 (0.9,4.2)	2.8 (1.5,4.5)	<0.001
BUN (mmol/L)	9.1 (5.6,17.1)	7.2 (4.9,13.9)	11.7 (6.6,19.1)	<0.001
HDL (mmol/L)	1.0 (0.8,1.3)	1.1 (0.9,1.3)	1.0 (0.8,1.2)	<0.001
LDL (mmol/L)	2.8 (2.2,3.7)	2.9 (2.3,3.9)	2.7 (2.2,3.6)	<0.001
TC (mmol/L)	4.5 (3.8,5.6)	4.7 (3.9,5.8)	4.4 (3.7,5.4)	<0.001
TG (mmol/L)	1.8 (1.3,2.5)	1.8 (1.2,2.6)	1.8 (1.3,2.5)	0.504
ALB (g/L)	34.6 (28.7,39.0)	35.3 (28.2,40.1)	33.9 (28.9,37.9)	<0.001
Na (mmol/L)	142.0 (139.5,144.6)	141.8 (139.4,144.2)	142.1 (139.7,145.3)	<0.001
K (mmol/L)	4.0 (3.7,4.4)	4.0 (3.7,4.3)	4.1 (3.8,4.5)	<0.001
lv(mmol/L)	104.2 (101.5,106.6)	104.3 (101.6,106.8)	104.2 (101.5,106.5)	0.014
Ca (mmol/L)	2.0 (1.1,2.2)	2.0 (1.1,2.2)	2.0 (1.1,2.2)	0.283
Mg (mmol/L)	0.9 (0.8,0.9)	0.9 (0.8,0.9)	0.9 (0.8,1.0)	<0.001
P (mmol/L)	2.8 (1.4,58.2)	2.2 (1.3,57.4)	8.5 (1.5,59.2)	<0.001
NLR	2.4 (1.7,3.7)	2.3 (1.6,3.4)	2.7 (1.8,4.0)	<0.001
MLR	0.2 (0.2,0.3)	0.2 (0.2,0.3)	0.2 (0.2,0.3)	<0.001
PLR	852.7 (631.9,1193.5)	838.0 (618.9,1179.3)	865.4 (642.4,1203.6)	<0.001
SII	538.2 (365.7,824.8)	525.7 (356.3,815.2)	551.1 (376.1,834.6)	<0.001
IL_6(pg/ml)	4.1 (2.1,8.6)	3.1 (2.0,6.4)	5.3 (2.9,10.6)	<0.001
CRP, n (%)	0.3 (0.1,0.5)	0.3 (0.1,0.3)	0.3 (0.1,0.8)	<0.001
CysC(mg/L)	2.0 (1.1,3.8)	1.5 (1.0,3.2)	2.5 (1.4,4.2)	<0.001
D-Dimer (ug/mL)	0.7 (0.4,1.5)	0.5 (0.3,1.2)	0.8 (0.5,1.8)	<0.001
APTT (s)	36.9 (34.1,40.2)	36.6 (33.8,39.9)	37.1 (34.4,40.5)	<0.001
PT (s)	13.3 (12.8,13.9)	13.2 (12.7,13.8)	13.4 (12.8,14.0)	<0.001
antiplatelet, n (%)	1544 (11.6)	1144 (17.2)	400 (6.0)	<0.001

BMI, body mass index; Hb, hemoglobin; Scr, serum creatinine; eGFR, estimated glomerular filtration rate; BUN, blood urea nitrogen; HDL, high-density lipoprotein; LDL, low-density lipoprotein; TC, total cholesterol; TG, triglycerides; ALB, albumin; NLR, neutrophil-to-lymphocyte ratio; MLR, Monocyte-lymphocyte count ratio; PLR, Platelet-lymphocyte count ratio; SII, systemic immune-inflammation index; IL_6, interleukin 6; CRP, Creactive protein; CysC, Cystatin C; D-Dimer, D-dimer; APTT, plasma-activated partial thromboplastin time; PT, prothrombin time.

### 3.3 Feature selection

**Figure 2:**
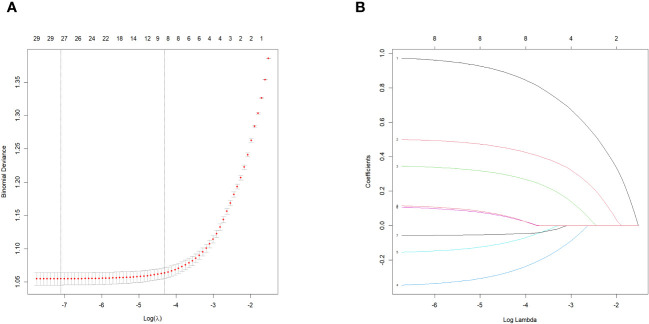
Features selection using the LASSO binomial regression model. LASSO, least absolute shrinkage and selection operator. **(A)** The partial likelihood deviance (binomial deviance) curve was plotted versus log (lambda).LASSO coefficient profiles of the 31 baseline features. **(B)** Tuning parameter **(A)** selection in the LASSO model used 5-fold cross validation via minimum criteria variable selection. LASSO coefficient profiles of the 8 features.

LASSO regression was used to select the important variables associated with CVD. The optimal parameter (lambda) in the LASSO regression model was determined using five-fold cross-validation. A dotted vertical line was drawn at the value of lambda that represents the best trade-off according to the minimum criterion, and another at the most regularized model within one standard error of the minimum (**Figure 2A**), while a vertical line was drawn at the value selected by five-fold cross-validation, where the optimal lambda produced eight features with non-zero coefficients (**Figure 2B**). Eight variables were found to be predictors of CVD occurrence (**Figure 2**), with the corresponding model risk factors being age, history of hypertension, sex, antiplatelet medication, HDL, sodium, 24-h urinary protein, and eGFR.

### 3.4 Model construction and evaluation

**Figure 3:**
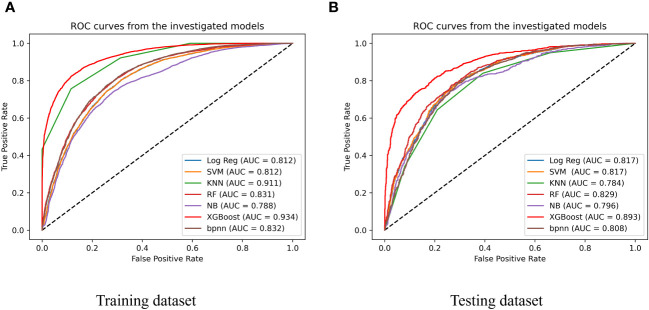
Performance of 7 types of predicting models of training dataset **(A)** and testing dataset **(B)**; SVM, support vector machine; Log Reg, logistic regression; XGBoost, extreme gradient boosting; KNN, k-nearest neighbor neighbor; NB, naïve Bayesian; RF, Random Forest; BPNN, Backpropagation Neural Network.

We evaluated seven machine-learning models for predicting CVD using training and testing datasets, including the SVM, LR, NB, KNN, XGBoost, RF, and BPNN to evaluate model performance. The training set was used to train the models, and the testing set was used to test their accuracy and generalizability. The performances of the different models is shown in **Figure 3** and **Table 2**. The test set had the following AUC values: SVM algorithm, 0.817; LR model, 0.817; KNN, 0.784 (lowest AUC); RF, 0.829; BPNN, 0.808; and NB algorithm, 0.796; and XGBoost algorithm, 0.893. The XGBoost model had the highest AUC, which was significantly higher than that of the other models. This indicates a good ability to distinguish between the presence and absence of CVD. Besides, the XGBoost model exhibited the highest accuracy (0.806), specificity (0.8), and F1 score (0.806), which suggests that it is the best performing model among those listed. The ranking of this CVD prediction model as one of the best models indicates that it has strong predictive ability and can be used in clinical settings.

**Table 2:** Performance metrics for seven models in testing dataset.

Model	AUC	SE	SP	AC	F1-score
SVM	0.817	0.770	0.727	0.745	0.744
LR	0.817	0.773	0.726	0.748	0.748
NB	0.796	0.745	0.736	0.736	0.736
KNN	0.784	0.840	0.608	0.717	0.716
XGBoost	0.893	0.814	0.800	0.806	0.806
RF	0.829	0.776	0.734	0.751	0.751
BNPP	0.808	0.788	0.708	0.742	0.742

SE, sensitivity; SP, specificity; AC, accuracy.

### 3.5 Model interpretation

**Figure 4:**
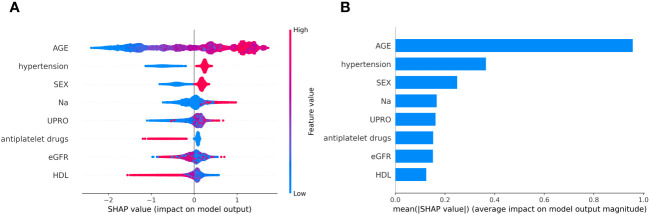
**(A)** SHAP summary plot in XGBoost model with 8 variables. **(B)** A importance matrix plot of the XGBoost.

**Figure 5:**
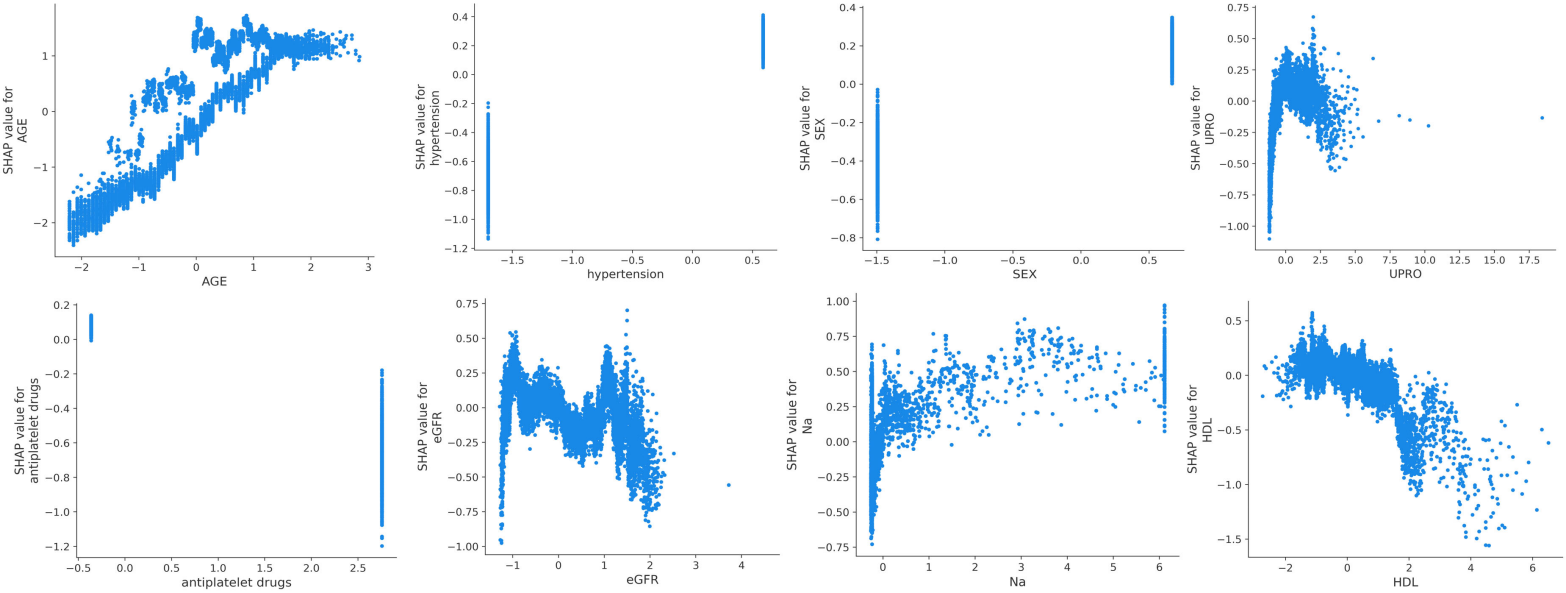
SHAP dependence plot of the XGBoost model.

**Figure 6:**
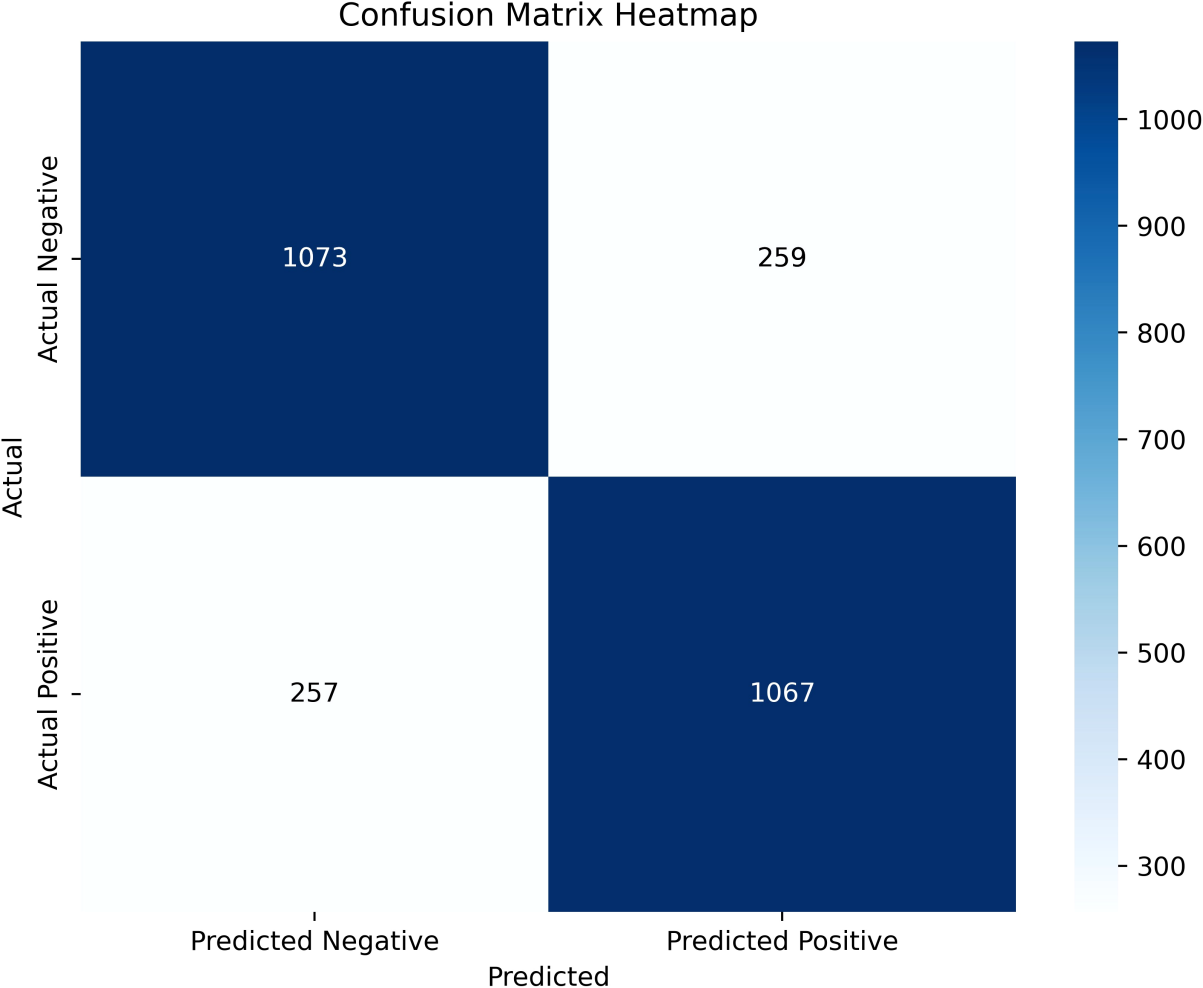
The confusion matrix of the XGBoost model predictions.

SHAP was used to interpret the predictions of the XGBoost machine learning model by calculating the contribution of each feature to the CVD prediction (14). **Figure 4B** shows the rankings of the top eight risk factors; the importance decreases with age, history of hypertension, sex, 24-h urinary protein, antiplatelet medication, eGFR, sodium, and HDL. Age was found to be the most influential feature, followed by history of hypertension and sex, which had the strongest predictive impact on the model. SHAP summary plots (**Figures 4A**, **5**) were used to visually represent the impact of each variable on the model’s output. The position of the SHAP value (x-axis) indicates the impact of the feature on prediction, with each point representing a sample, and the redder (bluer) color indicating higher (lower) feature values. If the SHAP value increases with an increase in the feature value, it indicates a positive correlation between the feature and the predicted outcome; otherwise, it indicates a negative relationship (**Figures 4A**, **5**). The results show that for age, history of hypertension, being male, and a lower GFR, many patients’ SHAP distributions are positive, indicating that an increase in age, having a history of hypertension, being male, and lower eGFR increase the risk of cardiovascular events. Higher levels of HDL and the use of antiplatelet medications reduce the risk of cardiovascular events. The prediction results of the XGBoost model are displayed using a confusion matrix, where the positive predictive value is 80.7% and the negative predictive value is 80.5% (**Figure 6**).

## 4 Discussion

This study retrospectively analyzed clinical data from the electronic medical records of 8,894 Chinese patients with CKD and successfully constructed a risk prediction model for the occurrence of CVD in these patients. To our knowledge, this is the first large-sample risk prediction model for cardiovascular events in CKD based on a Chinese population using clinical indicators, including adult patients with CKD. The demographic characteristics of these patients, including age and sex, were representative of patients with CKD, showing good model efficacy and a strong clinical application value.

Previous studies have explored the construction of prediction models for CVD in CKD (15–18). R. Avram et al. conducted a cohort study in which elastic net regression was employed to develop a proteomic risk model for predicting cardiovascular risk among 2,182 participants from a chronic kidney dysfunction cohort (19), with AUC values ranging from 0.84 to 0.89 over 1 to 10 years, yet the clinical model performed poorly, with AUCs between 0.70 and 0.73. In addition, the Chronic Renal Insufficiency Cohort study constructed a 10-year atherosclerotic cardiovascular disease risk prediction model for patients with CKD( (20)); the AUC of the Chronic Renal Insufficiency Cohort model developed using clinically available variables was 0.760, which targeted atherosclerotic cardiovascular disease and not composite cardiovascular events. A cohort study aimed to predict atrial fibrillation events in CKD with models developed using machine learning methods in the CKD population, which were compared to previously published prediction models; however, the C-index of the model using clinical variables was only 0.67 (7).

This study utilized clinical variables to construct a risk prediction model for cardiovascular events in CKD with superior performance. Among the seven machine learning models, most artificial intelligence models have shown a higher predictive performance than traditional LR and Cox regression models. Artificial Neural Networks (21) are highly suitable for extensive datasets rich in sequential and unstructured features, requiring the estimation of a large number of parameters, and thus necessitating substantial data to avoid overfitting. Machine learning, using routine clinical data, can accurately predict CVD in CKD. For this study, which primarily involves straightforward numerical variables, simpler machine learning models would be more appropriate. XGBoost, developed from RF, is not affected by multicollinearity and is characterized by its flexibility and efficiency.

In our study, we compared the performance of prediction models generated by seven machine learning algorithms and used the XGBoost ensemble machine learning method to construct the model. The results showed that the model had the highest AUC (0.89), sensitivity, and F1 score, indicating a good predictive effect on the risk of CVD in patients with CKD. This may be due to the effectiveness of XGBoost in handling complex patterns for disease prediction, outperforming other models.

Machine learning is often referred to as a “black box.” To explain the decision-making process of the XGBoost model algorithm, we employed the SHAP visualization method to interpret our predictions (14). The combination of machine learning and SHAP can provide clear explanations for individualized risk predictions and allow doctors to intuitively understand the impact of key features in the model (22). This study explained the contribution of the model and risk factors using SHAP values, which showed that age, hypertension, sex, and dyslipidemia can determine the risk of CVD in patients with CKD. Specifically, patients with CKD who are older, male, and have hypertension, lower eGFR, and lower high-density lipoprotein levels have a higher risk of CVD. The risk factors mentioned in the prediction model can be used to predict the CVD risk. This finding supports previous research and emphasizes the importance of risk factors for the occurrence of CVD events later in life. Starting from the mid-20th century with the Framingham Study (23), previous research has proven that older age, hypertension, male sex, and dyslipidemia are traditional independent risk factors for CVD in patients with CKD (24–27), which were also factors included in the Framingham prediction model (28). Sodium accumulates in tissues, potentially causing systemic inflammation and directly affecting myocardial and vascular structures. High sodium levels lead to blood pressure changes and sodium retention in patients with CKD, thereby increasing the risk of CVD (29). Compared with individuals without CKD, higher sodium content in the muscles and skin was observed in patients undergoing dialysis (30), which was positively correlated with systemic inflammation.

Previous research has identified multiple risk factors for CVD, while recent studies have focused on using artificial intelligence or regression-based models to identify new risk factors and provide insights into disease mechanisms, thereby improving the accuracy of CVD predictions in patients with CKD. These computational models attempt to overcome the limitations of the traditional models by incorporating a broader range of variables and using advanced techniques. The integration of novel CKD-specific markers and the use of complex computational techniques are expected to resolve this issue. The new models combine traditional and CKD-specific risk factors, recognizing the complex interactions between CKD progression and cardiovascular health. Many studies have confirmed that a decline in eGFR and albuminuria are independent risk factors for an increased risk of CVD death (31, 32). Kunihiro Matsushita et al (15) added unique kidney indicators, such as eGFR and the urine albumin–creatinine ratio (UACR) to the CKD supplement model, verifying that their inclusion significantly improves the risk prediction of CVD in patients with CKD. This is also relevant as the primary clinical guidelines for CVD prevention are yet to adopt CKD in CVD risk prediction; SCr is recommended as a primary marker of the eGFR in the current KDOQI guidelines, and eGFR-defined CKD is related to adverse CVD outcomes (33). This study incorporated 24-h proteinuria into the model, which differs from the previously used albuminuria and the UACR. Considering the higher cost of albumin measurement compared to total protein measurement, using UACR or urine protein–creatinine ratio for population screening is reasonable (33). However, despite the convenience of urine protein–creatinine ratio (UPCR) and UACR in quantifying proteinuria, their use has limitations. The random measured UPCR or UACR may not always reflect the 24-h excretion rate because protein or albumin excretion varies with the time of the day, stress levels, fatigue, and other factors. Therefore, incorporating the 24-h quantitative measurement of proteinuria into models, as illustrated by SHAP graphs, shows that an increase in 24-h proteinuria can lead to an increased risk of CKD. As traditional CVD risk factors may have different weights in the prediction factors among the CKD population (34), other indicators can serve as valuable supplements to enhance the predictive capability of such models. SHAP visualizations provide information for clinical decision making, highlighting the complexity of predictive models. Factors included in the predictive model are rooted in established and emerging evidence. New indicators of kidney disease risk are integrated into relevant predictive models as complements, offering better possibilities for clinicians to accurately assess patients’ cardiovascular risk and take appropriate intervention measures to reduce the incidence of cardiovascular events.

This study has some limitations. First, the model was not validated externally and was based on retrospective data, necessitating prospective cohort studies to verify the accuracy and stability of the model. Second, only routine clinical indicators were included in this study, and the addition of novel biomarkers from multi-omics studies may further enhance the model. However, this study was based on a large sample of the Chinese population and had a good model effect. Additionally, the use of clinically accessible indicators to build a CVD prediction model has strong clinical application value.

Moving forward, we may consider integrating our predictive model into clinical practice via mini-programs and mobile applications. This approach may facilitate precise diagnosis and treatment in clinical settings.

In conclusion, this study successfully established a risk-prediction model for CVD in patients with CKD. The risk prediction model is expected to serve as a practical tool for clinicians to identify high-risk individuals at an early stage and initiate targeted interventions in a timely manner, thus improving the scientific accuracy of clinical decision-making.

## Data availability statement

The original contributions presented in the study are included in the article/**Supplementary Material**. Further inquiries can be directed to the corresponding authors.

## Ethics statement

All methods were performed in accordance with the Declaration of Helsinki and the relevant guidelines and regulations. As we used anonymized and deidentified data, the need for written informed consent was waived by the Ethics Committee of the General Hospital of the Chinese People’s Liberation Army due to the retrospective nature of the study (approval number S2021-696-01).

## Author contributions

HZ: Data curation, Formal analysis, Validation, Writing – original draft, Writing – review & editing. SQ: Data curation, Formal analysis, Writing – review & editing. DZ: Conceptualization, Investigation, Methodology, Writing – review & editing. KW: Methodology, Project administration, Supervision, Validation, Writing – review & editing. BW: Formal analysis, Methodology, Supervision, Writing – review & editing. YN: Data curation, Formal analysis, Project administration, Writing – review & editing. SS: Methodology, Supervision, Writing – review & editing. ZD: Supervision, Writing – review & editing. WZ: Supervision, Writing – review & editing. YZ: Conceptualization, Data curation, Methodology, Project administration, Supervision, Writing – review & editing. XC: Investigation, Methodology, Project administration, Supervision, Writing – review & editing.
