# Supplementary figures and images for: Machine learning model for cardiovascular disease prediction in patients with chronic kidney disease
Source: Front Endocrinol (Lausanne). 2024 May 28;15:1390729. doi: 10.3389/fendo.2024.1390729 (PMC11165240; doi:10.3389/fendo.2024.1390729)

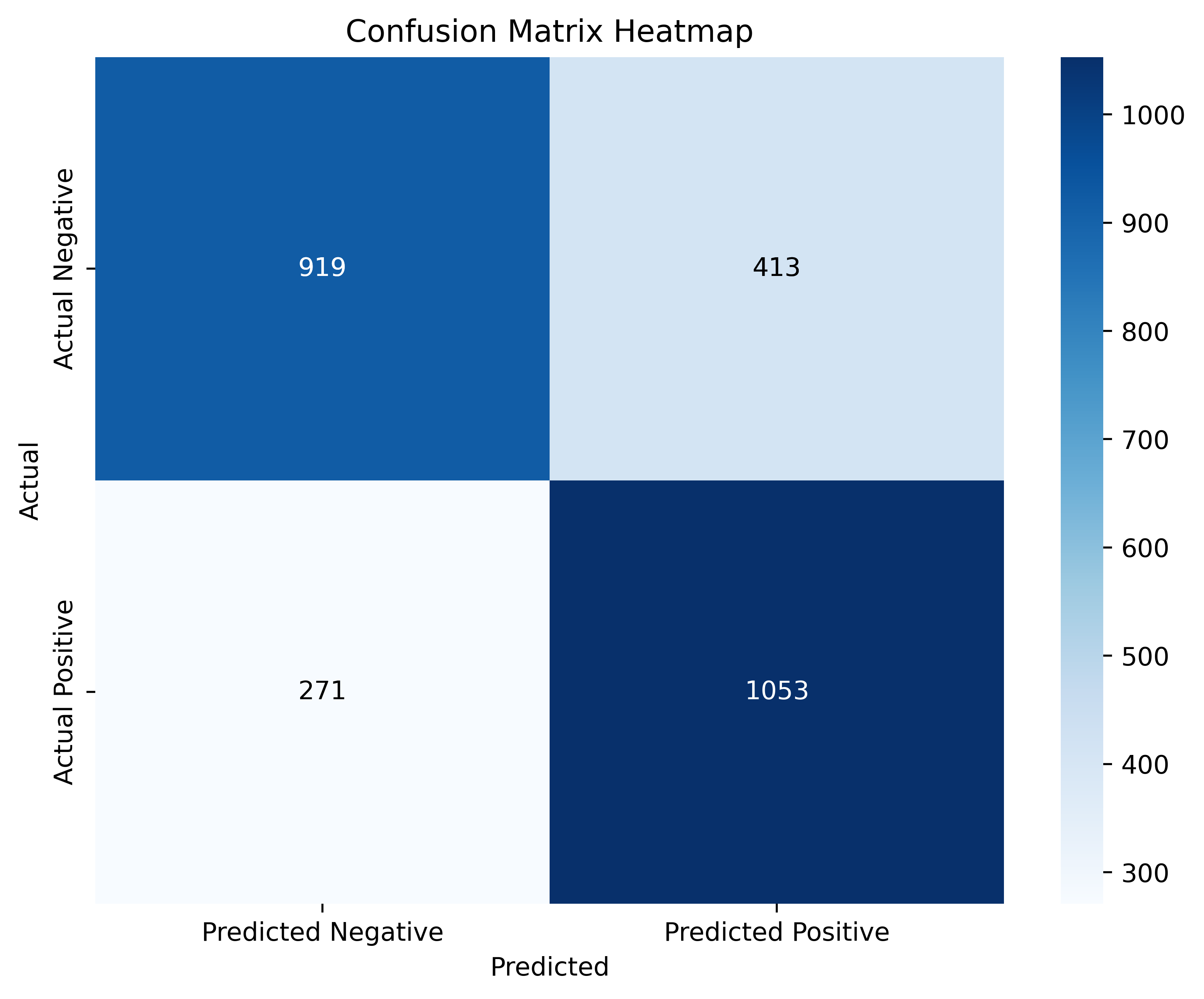

Supplement: Supplementary file 1 [file DataSheet_1.zip › supplementary materials/test_bpnn.tiff]

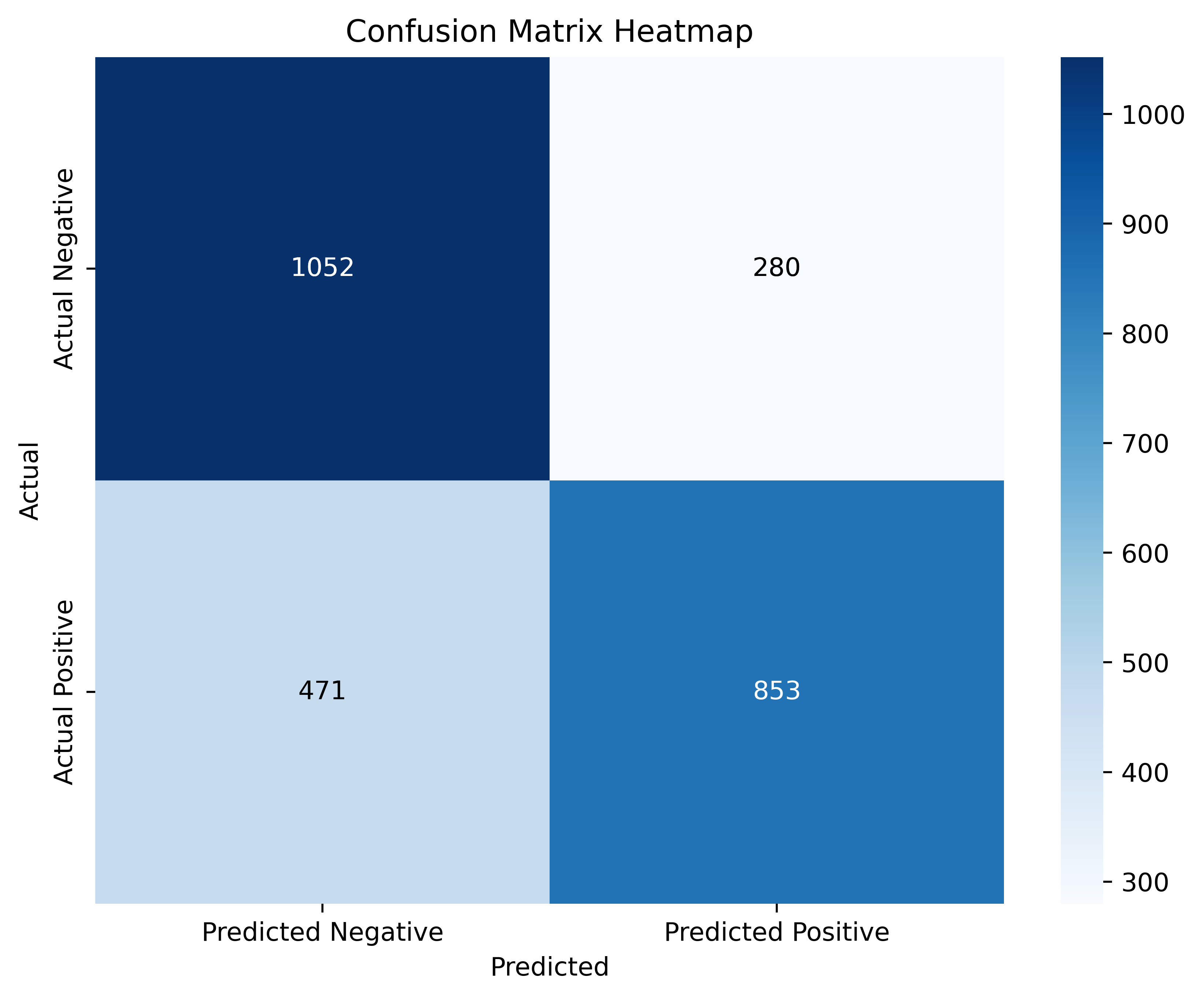

Supplement: Supplementary file 1 [file DataSheet_1.zip › supplementary materials/test_knn.tiff]

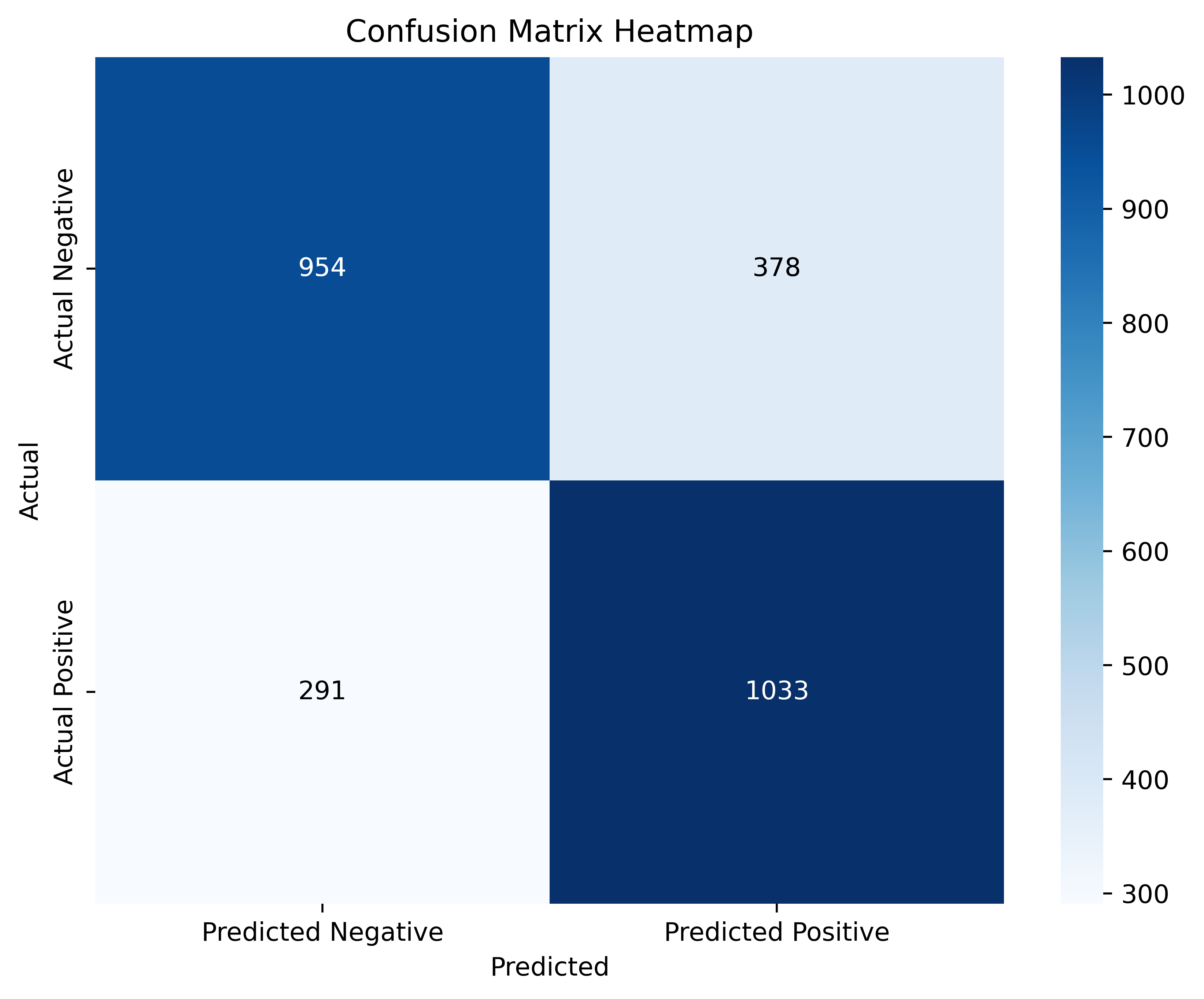

Supplement: Supplementary file 1 [file DataSheet_1.zip › supplementary materials/test_lr.tiff]

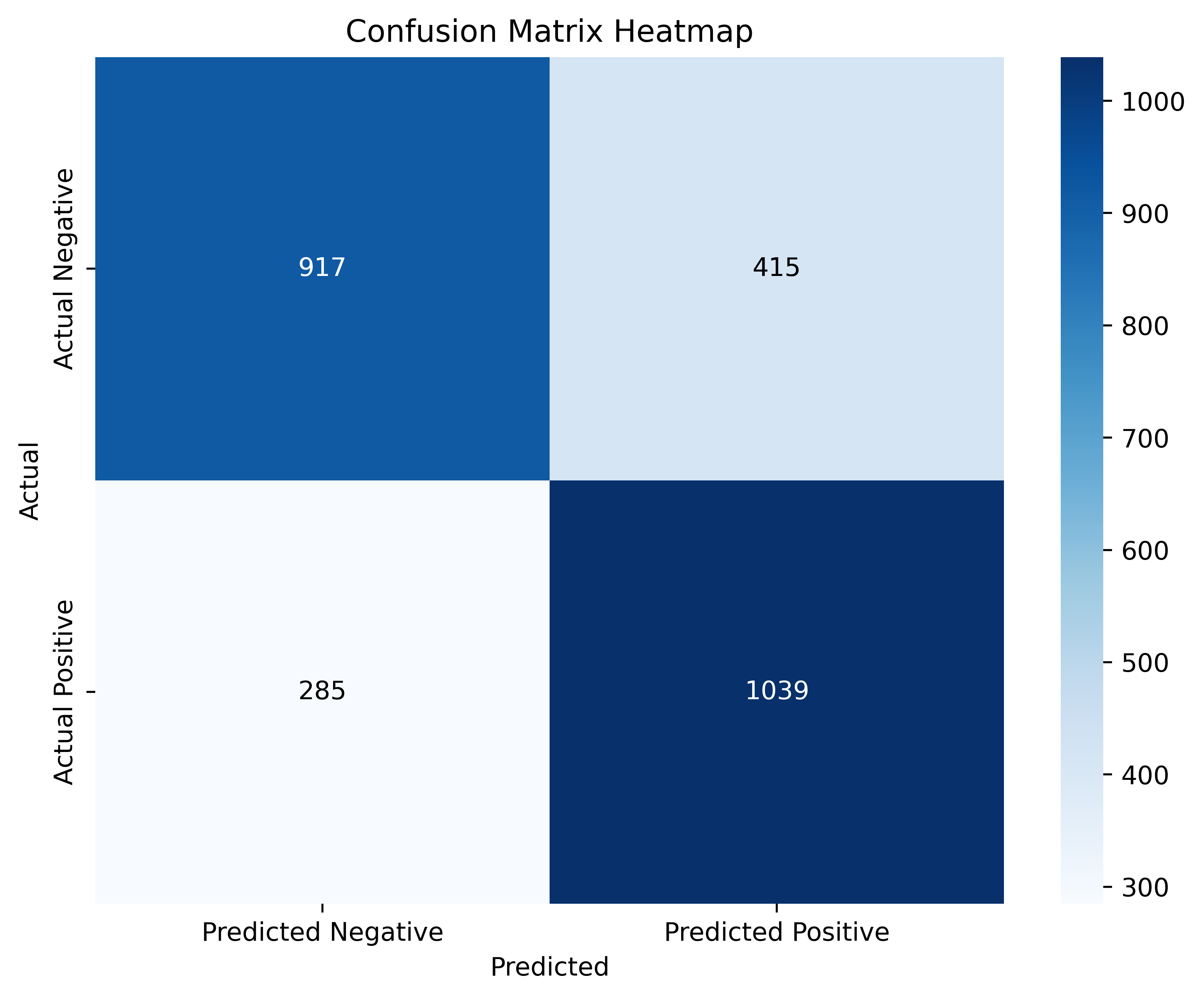

Supplement: Supplementary file 1 [file DataSheet_1.zip › supplementary materials/test_nb.tiff]

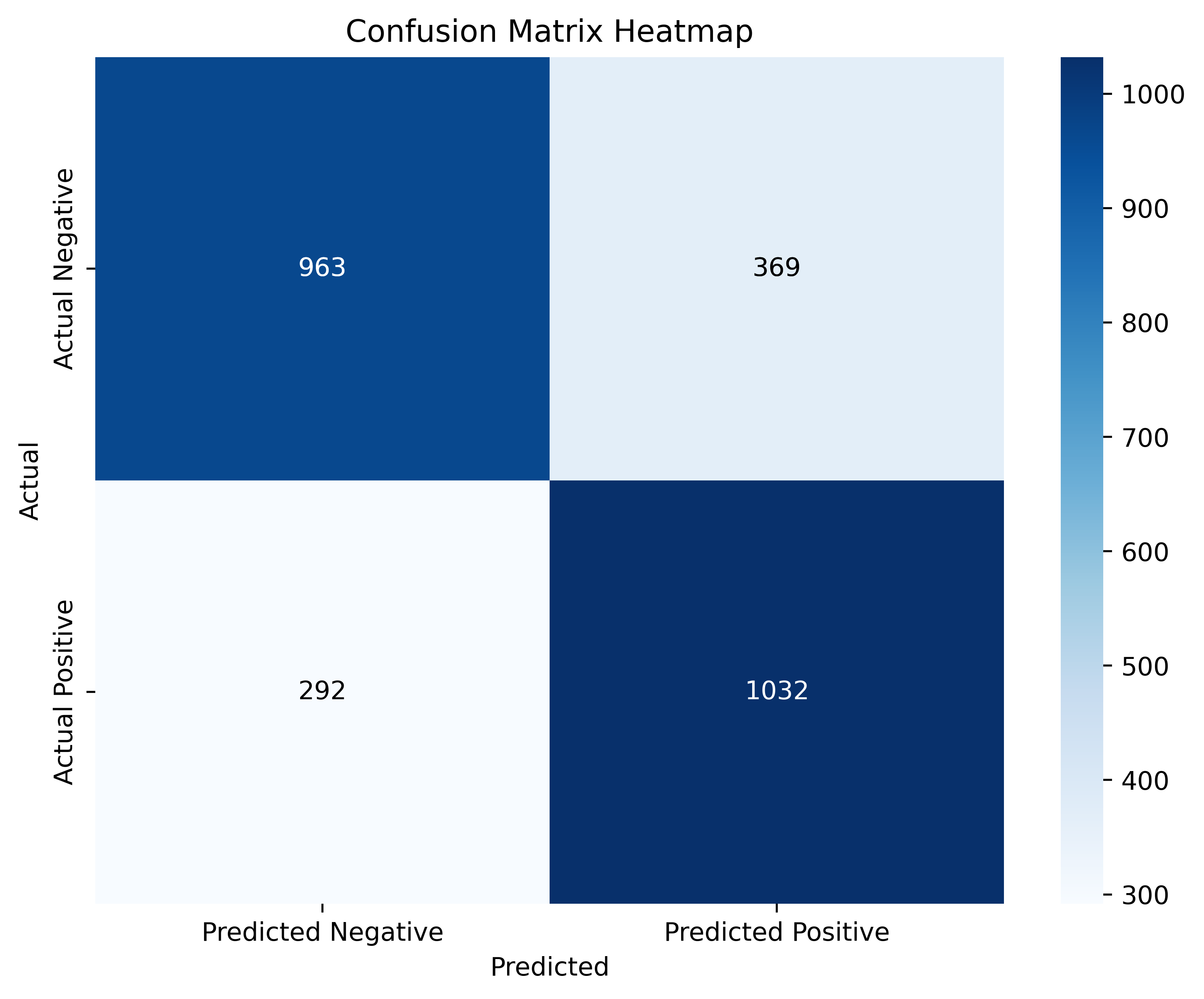

Supplement: Supplementary file 1 [file DataSheet_1.zip › supplementary materials/test_rf.tiff]

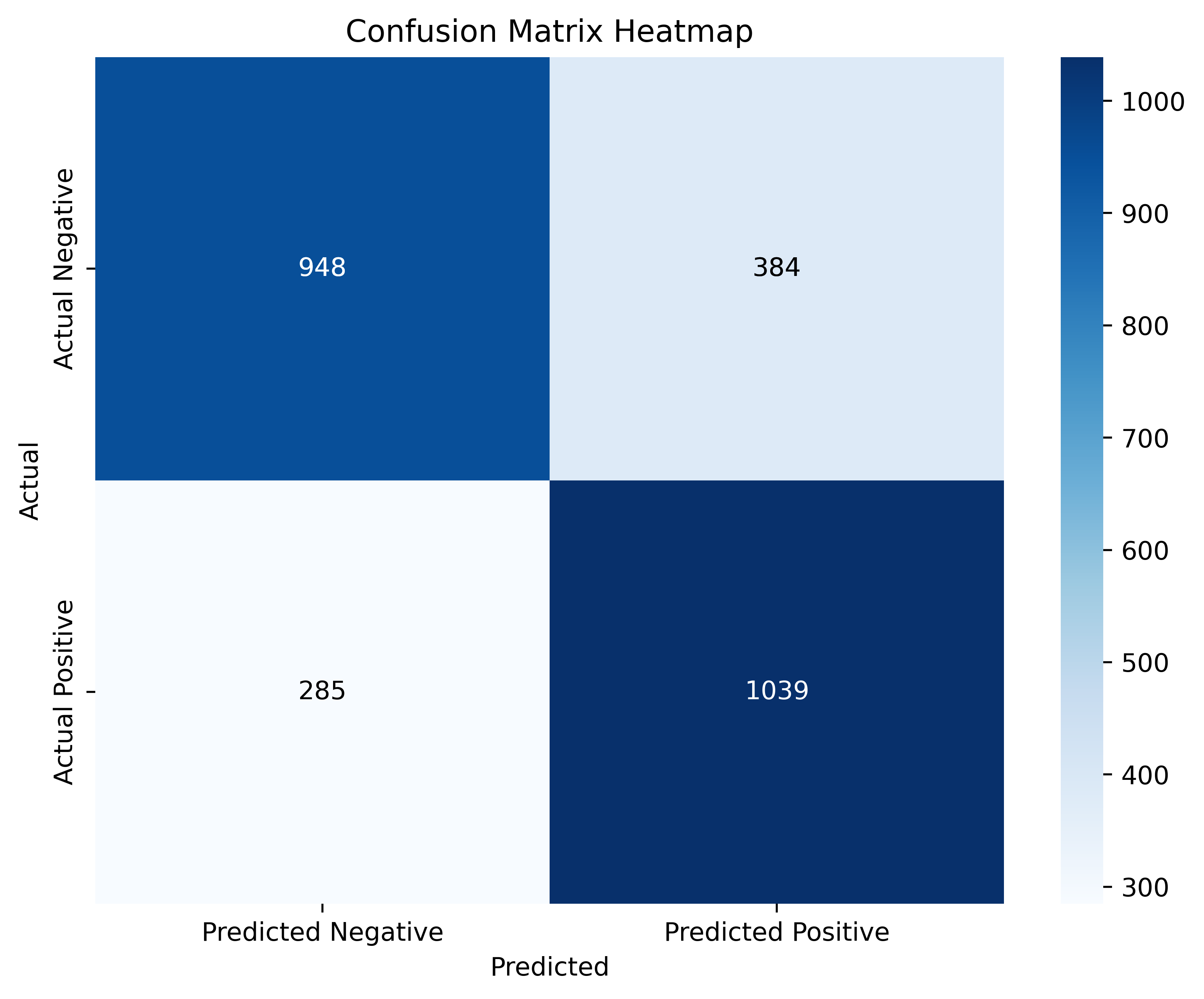

Supplement: Supplementary file 1 [file DataSheet_1.zip › supplementary materials/test_svm.tiff]

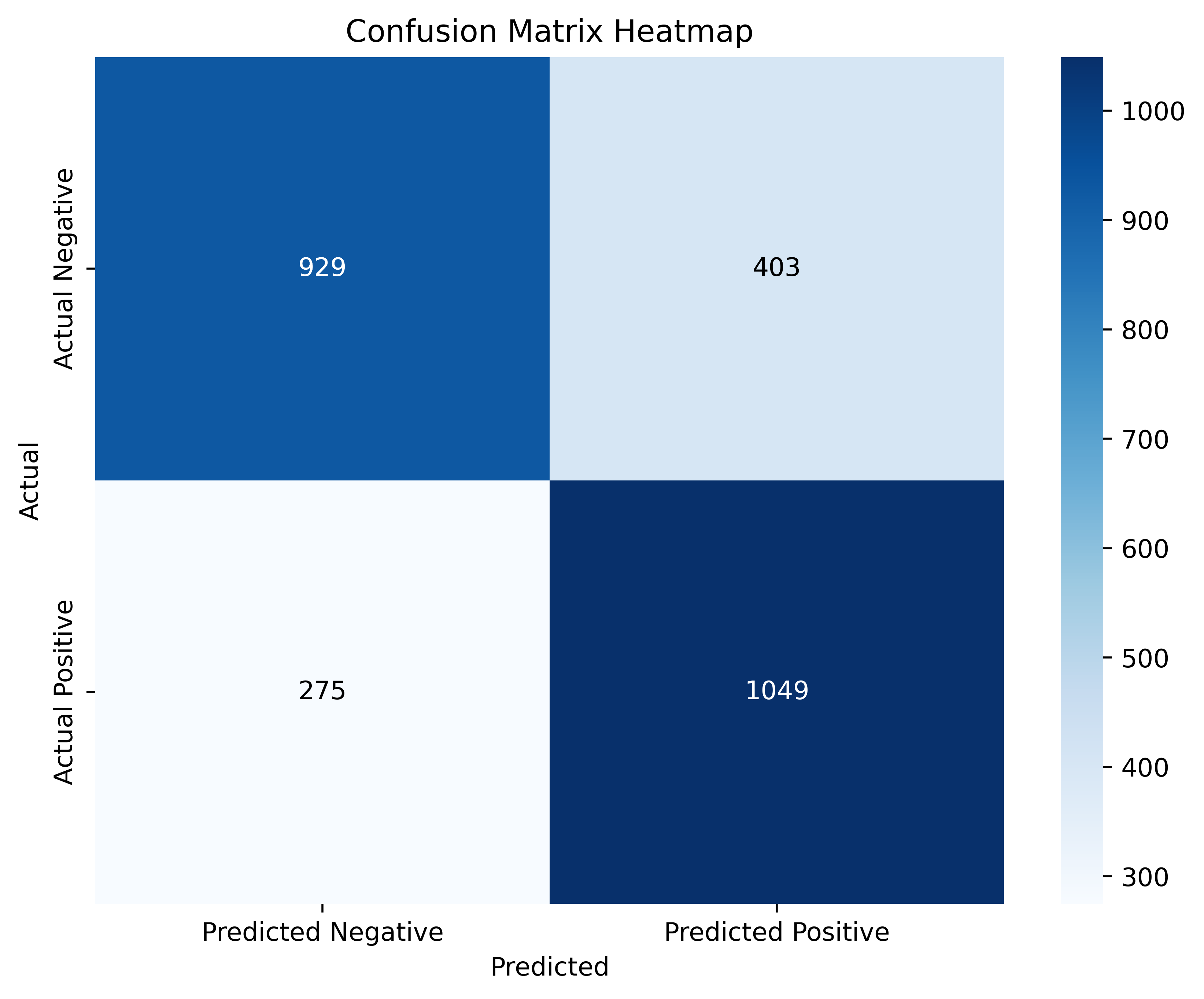

Supplement: Supplementary file 1 [file DataSheet_1.zip › supplementary materials/test_svmlinear.tiff]

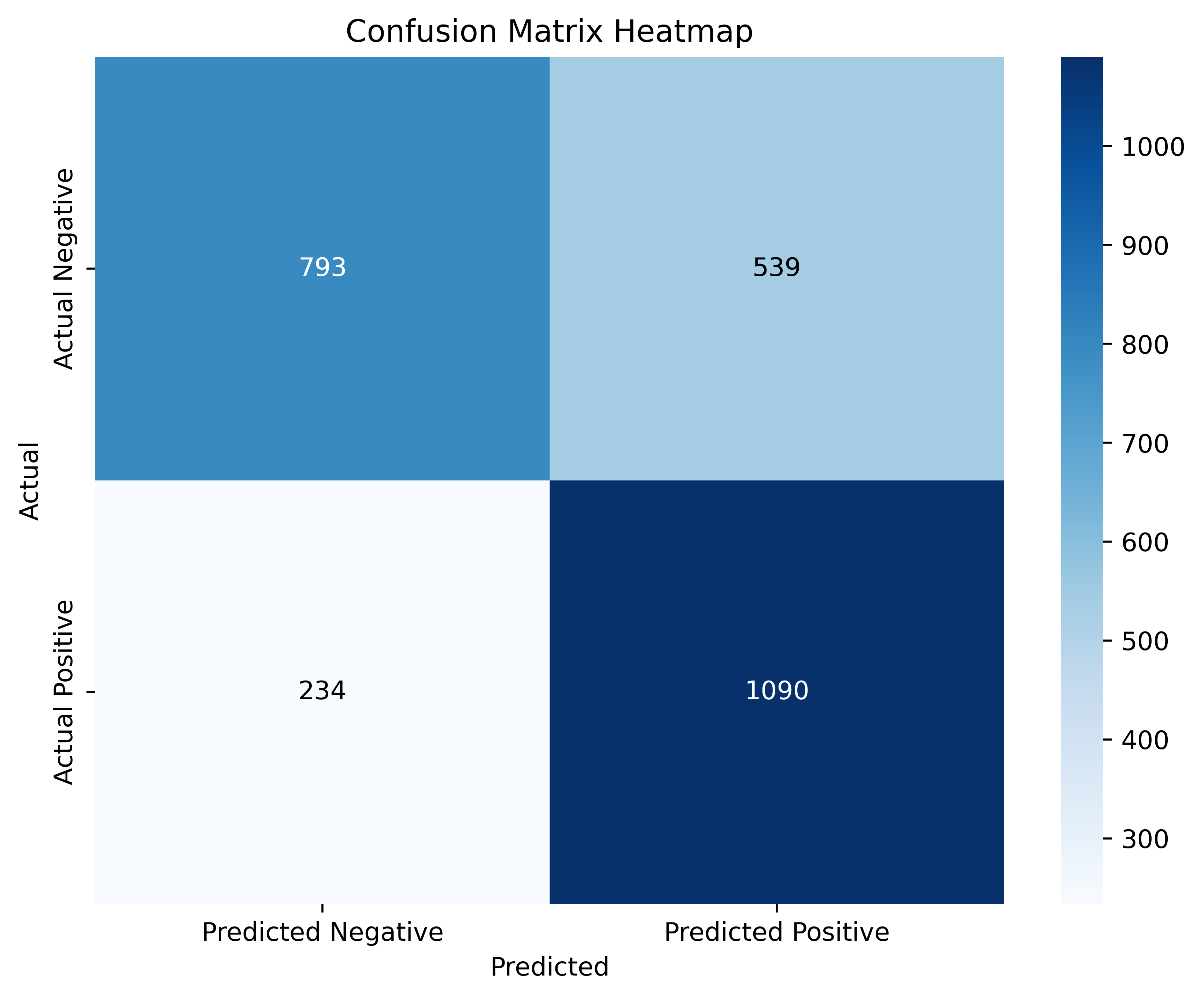

Supplement: Supplementary file 1 [file DataSheet_1.zip › supplementary materials/test_tree.tiff]

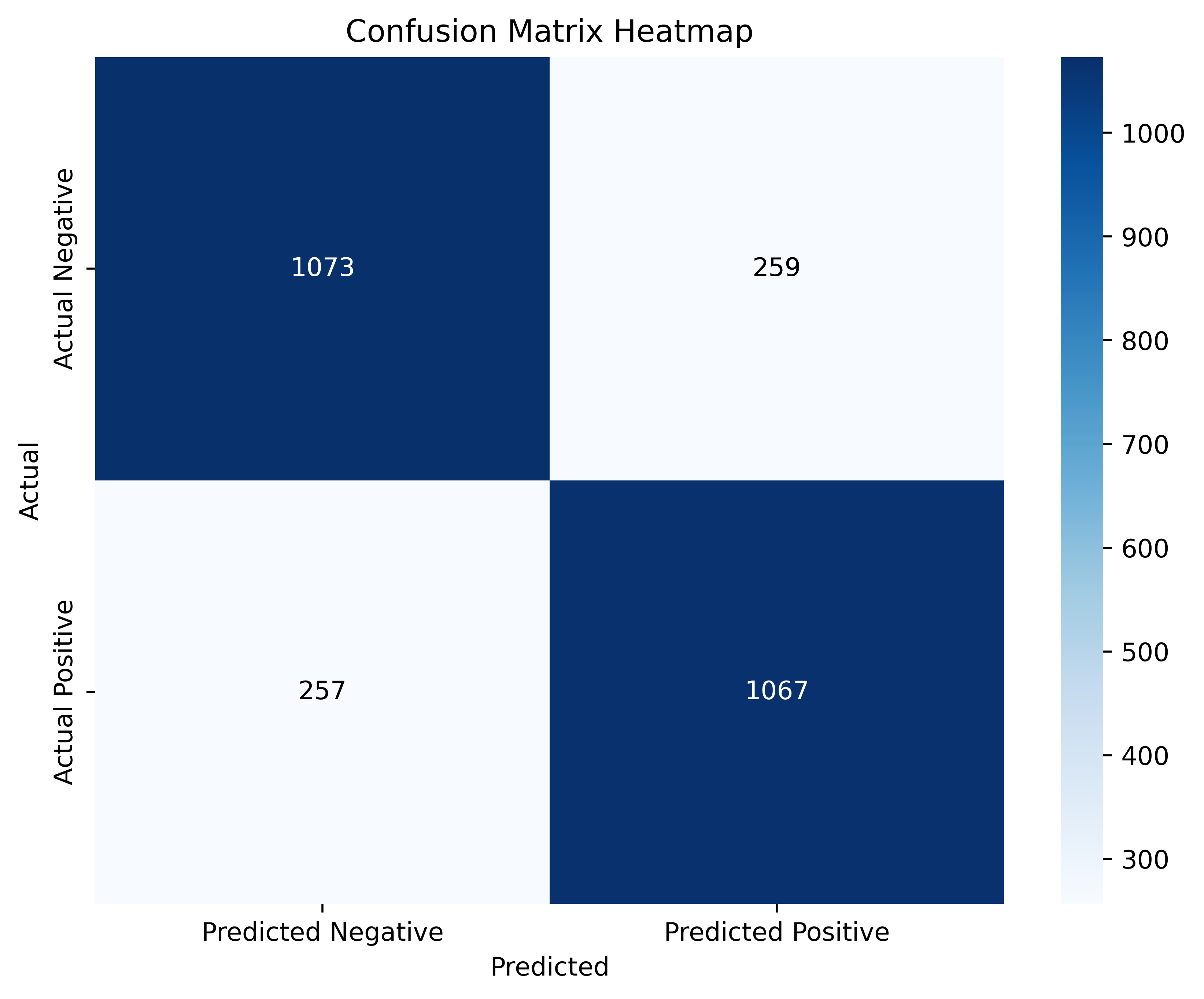

Supplement: Supplementary file 1 [file DataSheet_1.zip › supplementary materials/test_xgb.tiff]

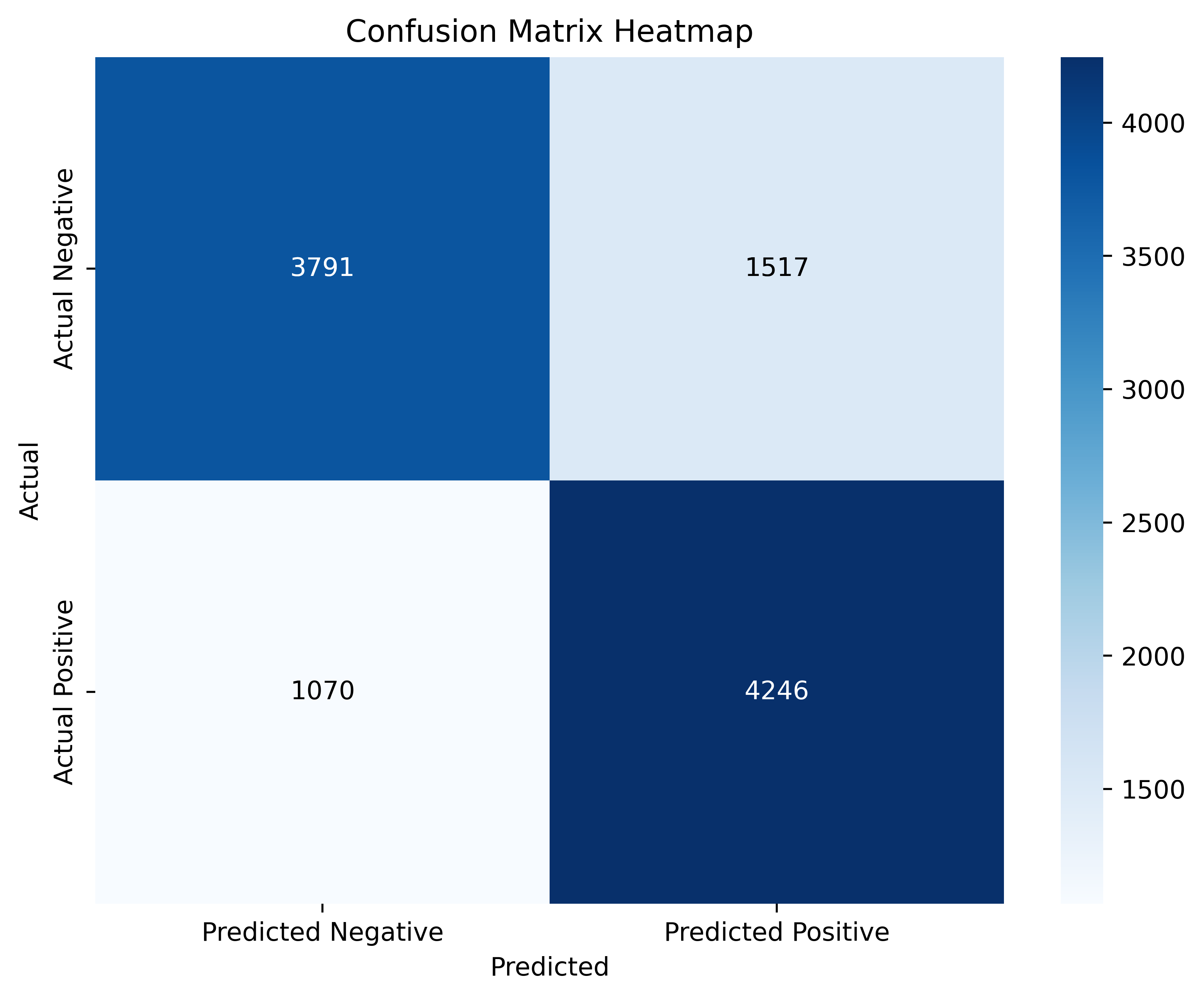

Supplement: Supplementary file 1 [file DataSheet_1.zip › supplementary materials/train_bpnn.tiff]

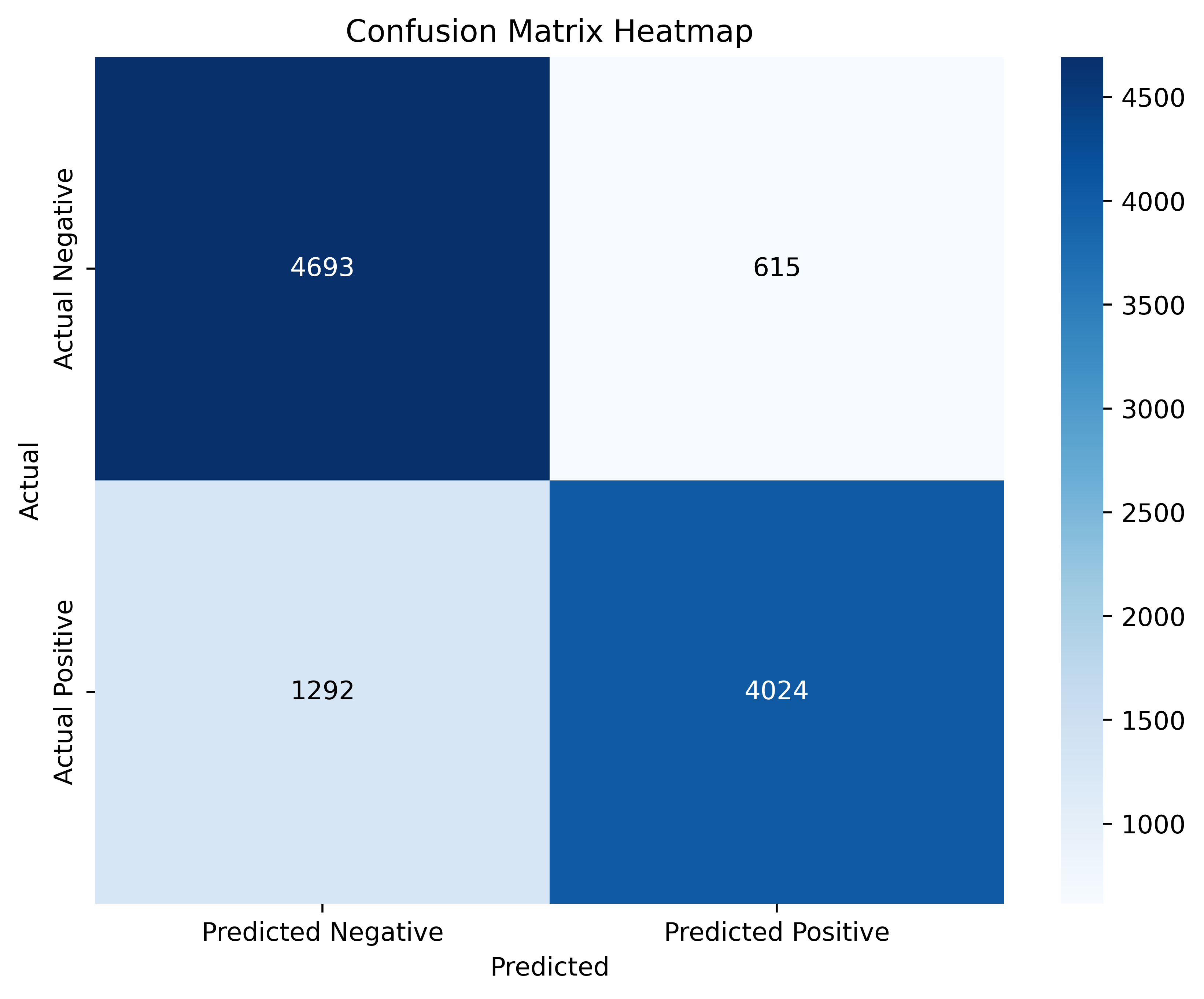

Supplement: Supplementary file 1 [file DataSheet_1.zip › supplementary materials/train_knn.tiff]

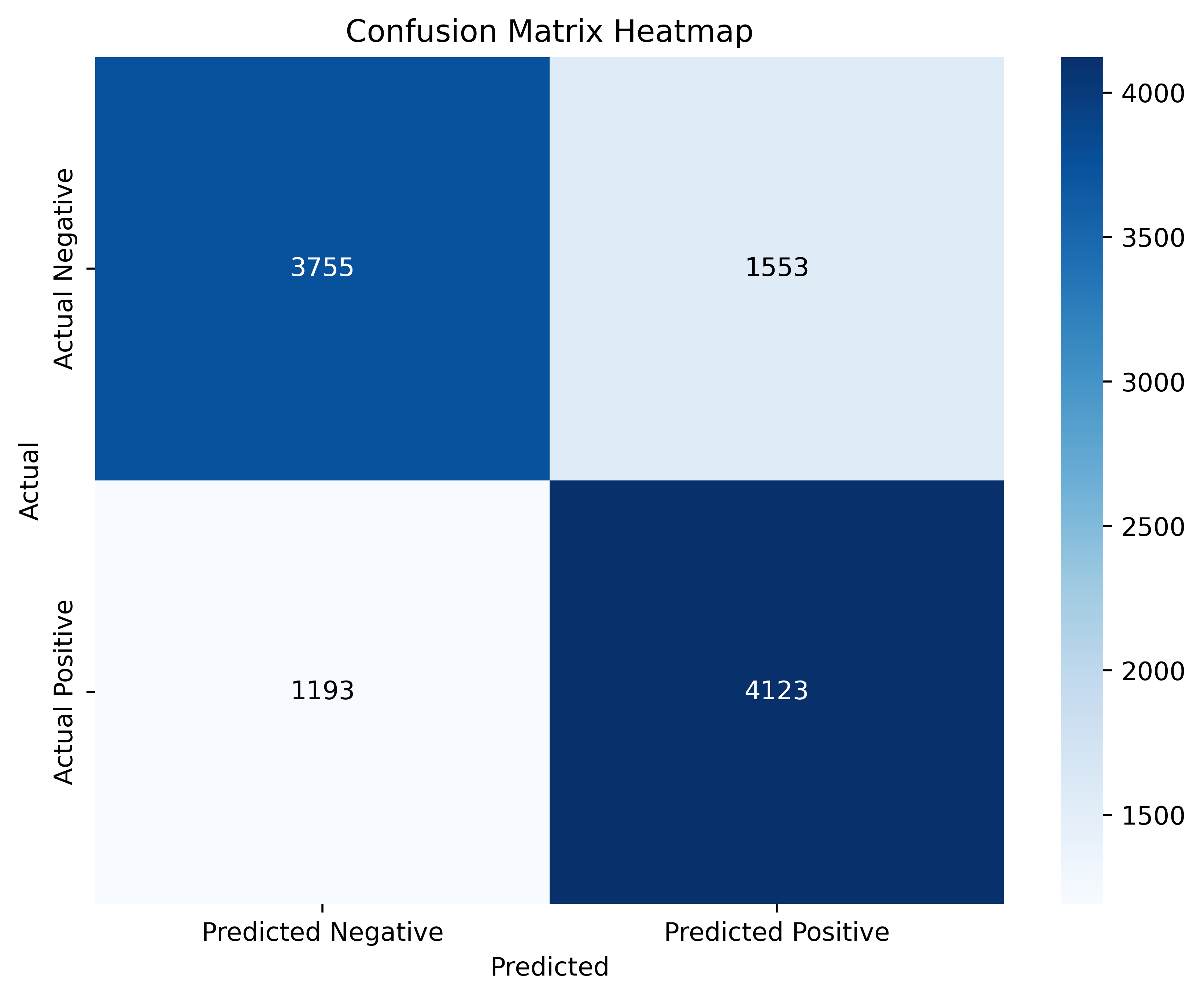

Supplement: Supplementary file 1 [file DataSheet_1.zip › supplementary materials/train_lr.tiff]

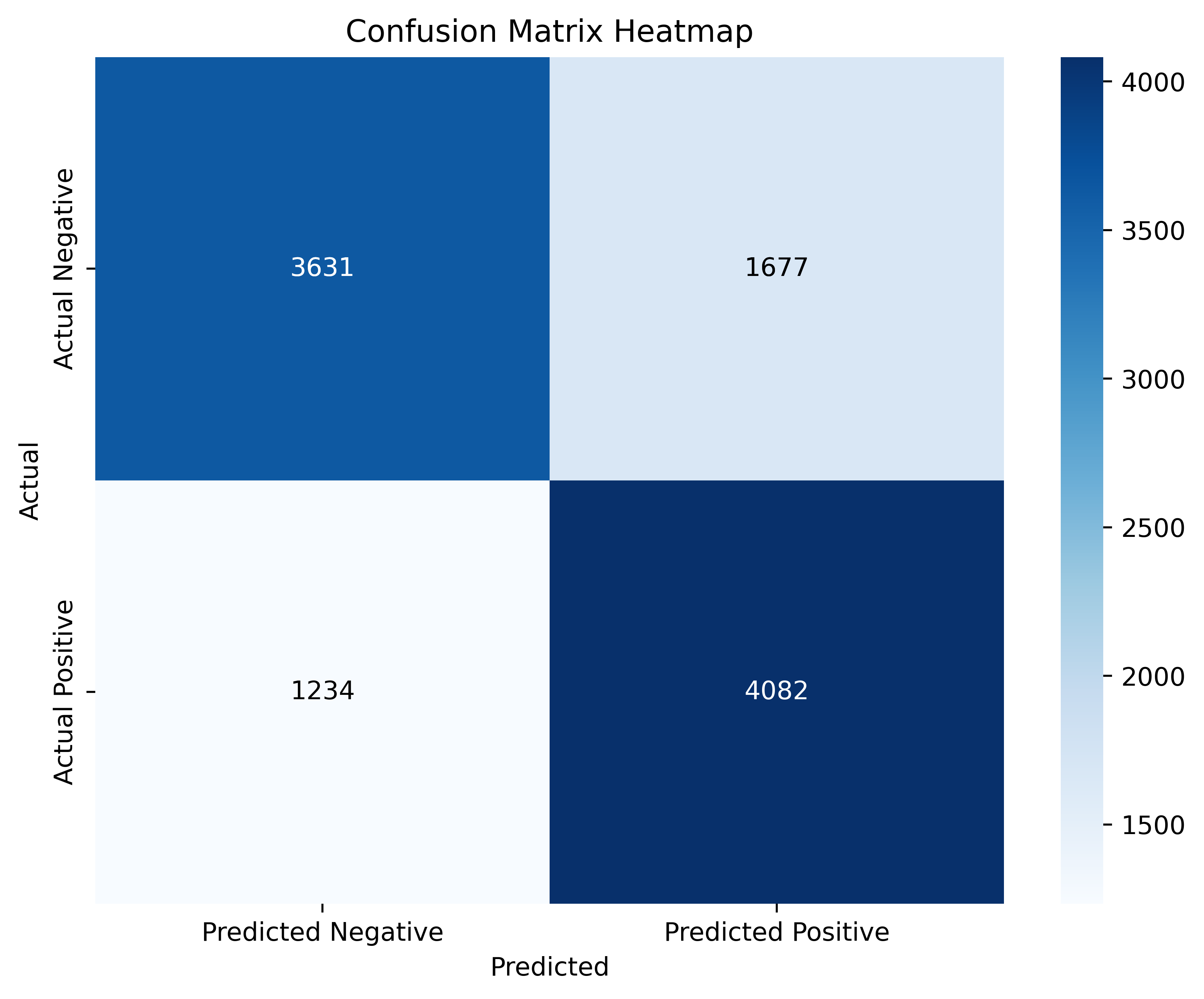

Supplement: Supplementary file 1 [file DataSheet_1.zip › supplementary materials/train_nb.tiff]

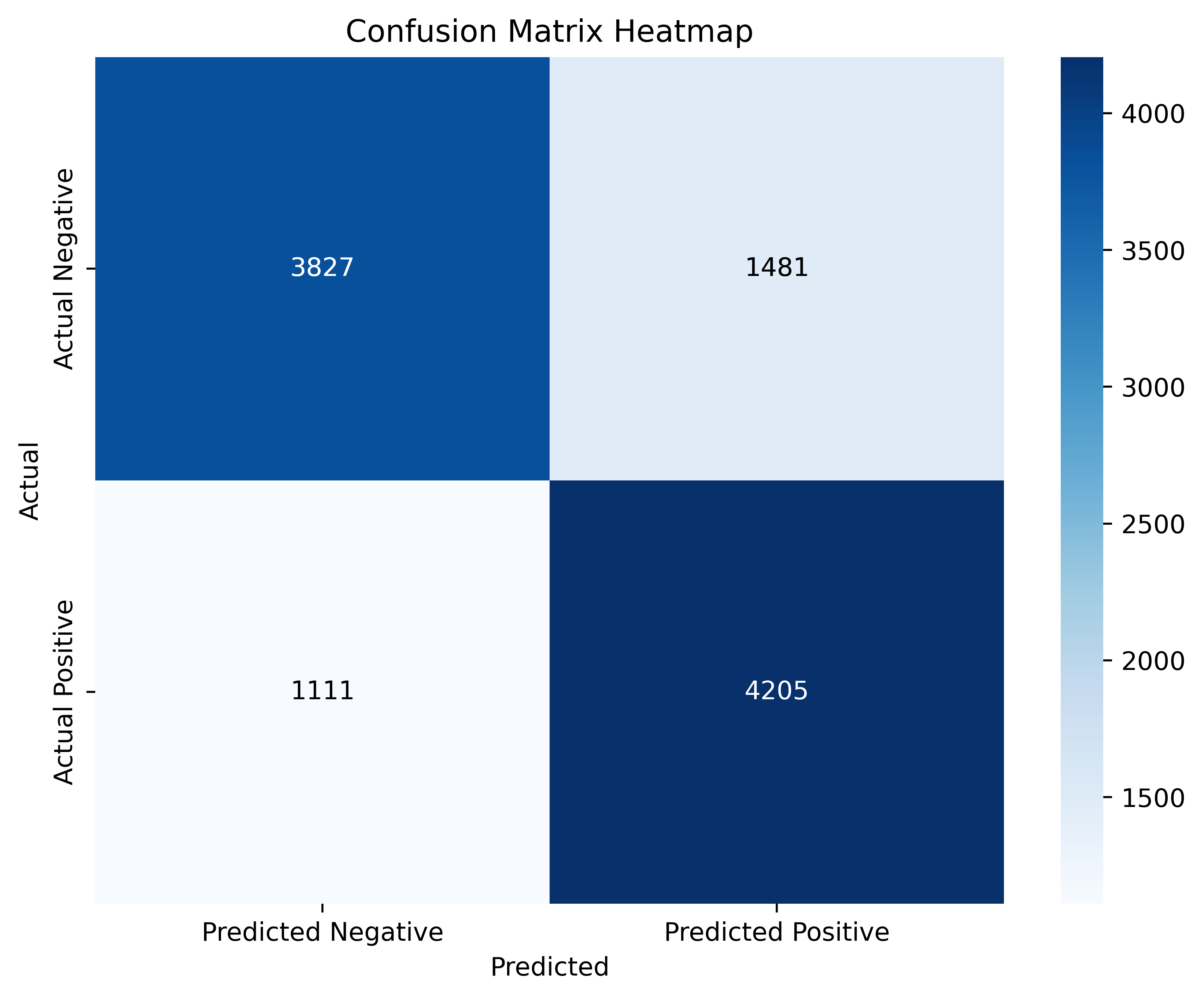

Supplement: Supplementary file 1 [file DataSheet_1.zip › supplementary materials/train_rf.tiff]

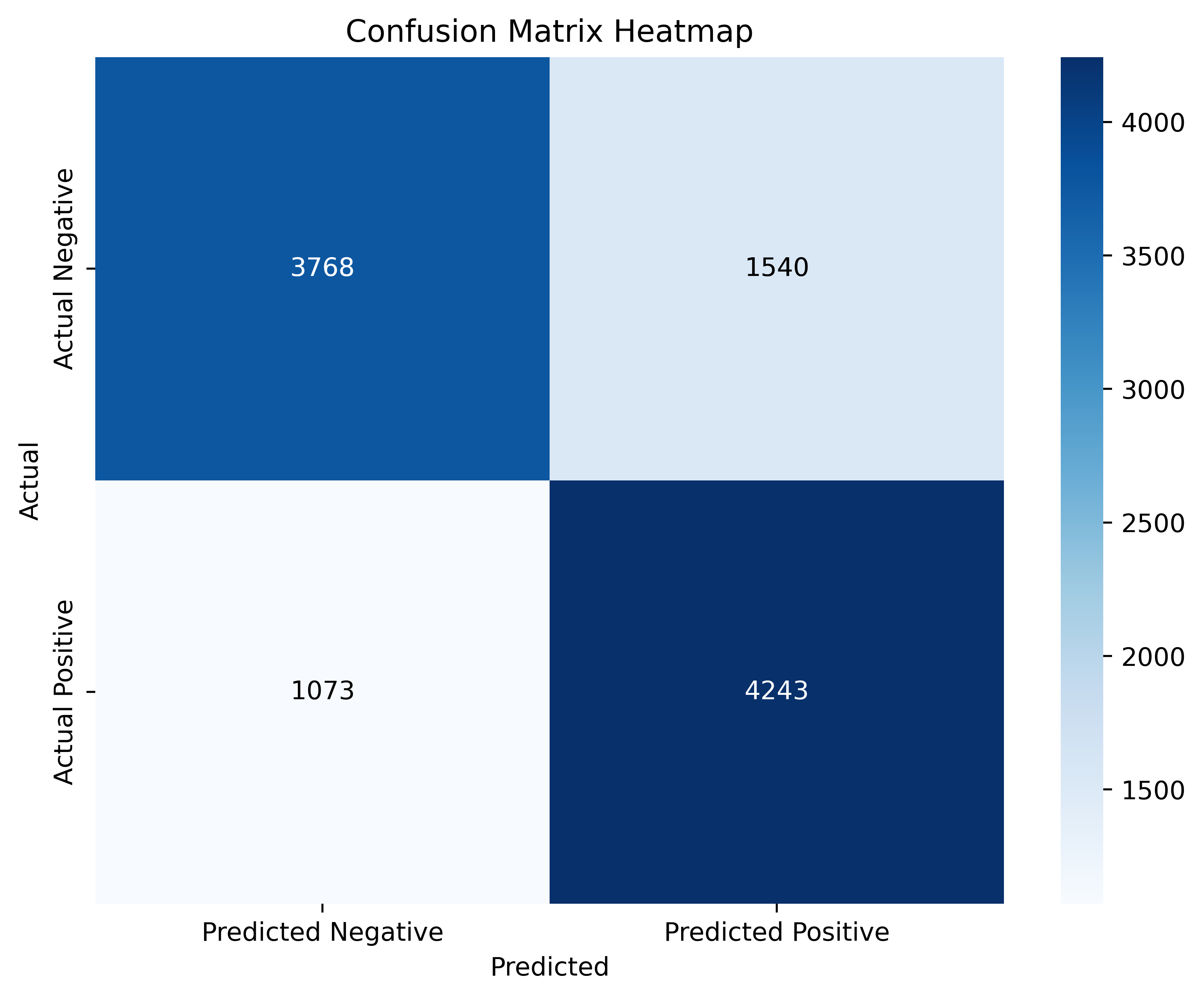

Supplement: Supplementary file 1 [file DataSheet_1.zip › supplementary materials/train_svm.tiff]

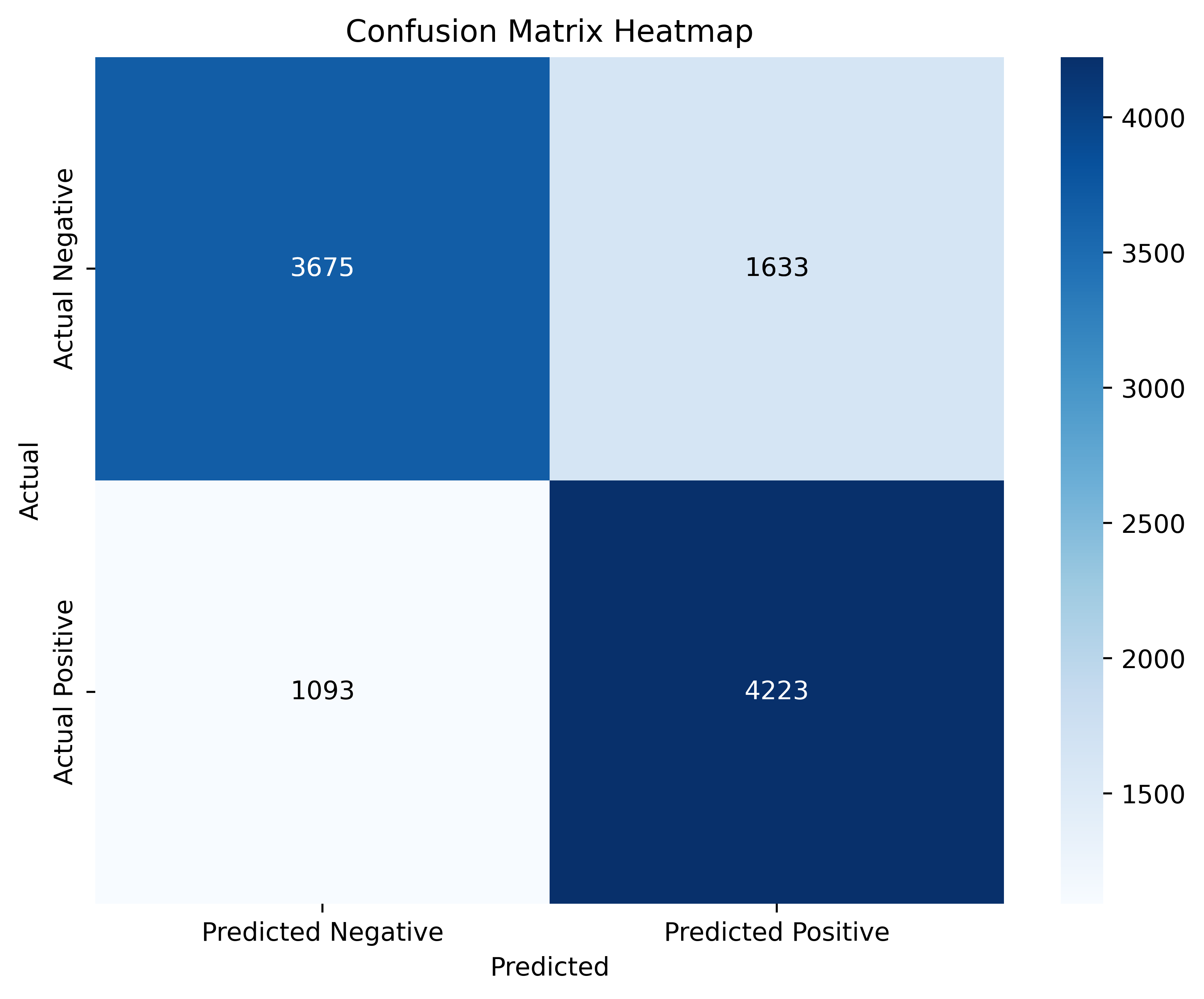

Supplement: Supplementary file 1 [file DataSheet_1.zip › supplementary materials/train_svmlinear.tiff]

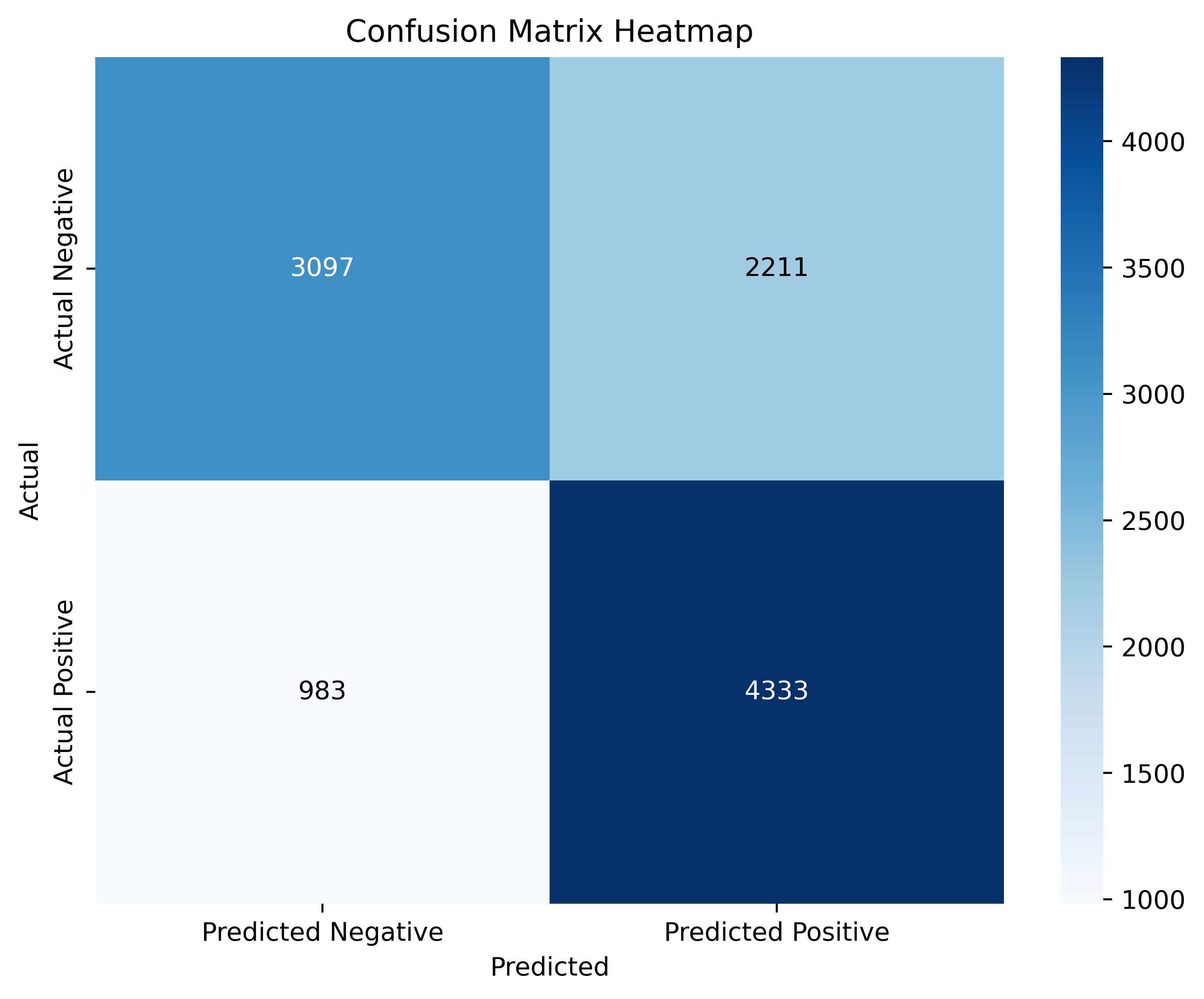

Supplement: Supplementary file 1 [file DataSheet_1.zip › supplementary materials/train_tree.tiff]

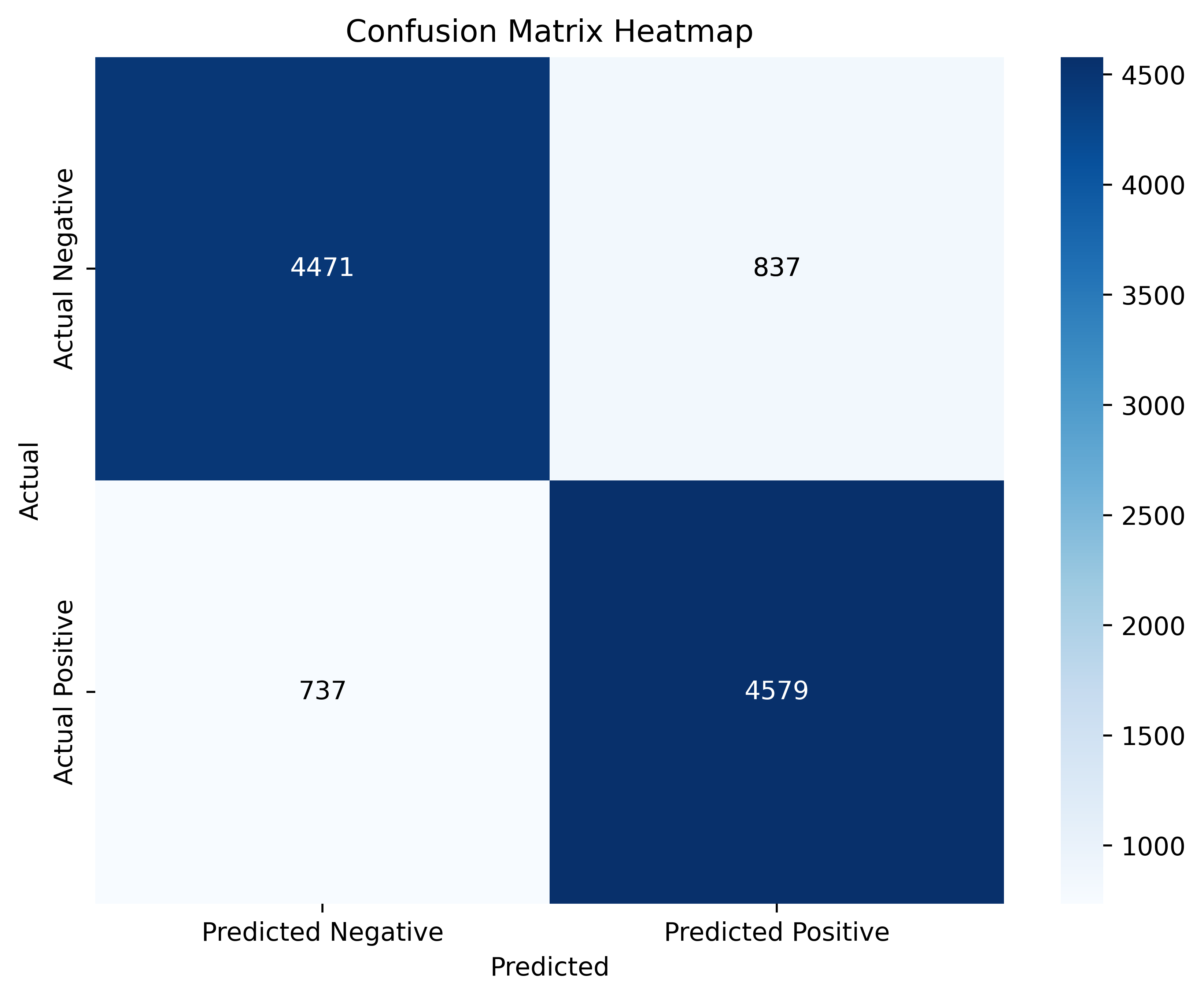

Supplement: Supplementary file 1 [file DataSheet_1.zip › supplementary materials/train_xgb.tiff]
